# Monocyte-Derived Signals Activate Human Natural Killer Cells in Response to *Leishmania* Parasites

**DOI:** 10.3389/fimmu.2018.00024

**Published:** 2018-01-24

**Authors:** Helena Messlinger, Heidi Sebald, Lukas Heger, Diana Dudziak, Christian Bogdan, Ulrike Schleicher

**Affiliations:** ^1^Mikrobiologisches Institut – Klinische Mikrobiologie, Immunologie und Hygiene, Universitätsklinikum Erlangen, Friedrich-Alexander-Universität (FAU) Erlangen-Nürnberg, Erlangen, Germany; ^2^Laboratory of DC Biology, Department of Dermatology, Universitätsklinikum Erlangen, Friedrich-Alexander-Universität (FAU) Erlangen-Nürnberg, Erlangen, Germany; ^3^Medical Immunology Campus Erlangen, Friedrich-Alexander-Universität (FAU) Erlangen-Nürnberg, Erlangen, Germany

**Keywords:** *Leishmania*, natural killer cells, monocytes, innate immunity, human cutaneous and visceral leishmaniasis

## Abstract

Activated natural killer (NK) cells release interferon (IFN)-γ, which is crucial for the control of intracellular pathogens such as *Leishmania*. In contrast to experimental murine leishmaniasis, the human NK cell response to *Leishmania* is still poorly characterized. Here, we investigated the interaction of human blood NK cells with promastigotes of different *Leishmania* species (*Leishmania major, Leishmania mexicana, Leishmania infantum*, and *Leishmania donovani*). When peripheral blood mononuclear cells or purified NK cells and monocytes (all derived from healthy blood donors from Germany without a history of leishmaniasis) were exposed to promastigotes, NK cells showed increased surface expression of the activation marker CD69. The extent of this effect varied depending on the *Leishmania* species; differences between dermotropic and viscerotropic *L. infantum* strains were not observed. Upregulation of CD69 required direct contact between monocytes and *Leishmania* and was partly inhibitable by anti-interleukin (IL)-18. Unexpectedly, IL-18 was undetectable in most of the supernatants (SNs) of monocyte/parasite cocultures. Confocal fluorescence microscopy of non-permeabilized cells revealed that *Leishmania*-infected monocytes trans-presented IL-18 to NK cells. Native, but not heat-treated SNs of monocyte/*Leishmania* cocultures also induced CD69 on NK cells, indicating the involvement of a soluble heat-labile factor other than IL-18. A role for the NK cell-activating cytokines IL-1β, IL-2, IL-12, IL-15, IL-21, and IFN-α/β was excluded. The increase of CD69 was not paralleled by NK cell IFN-γ production or enhanced cytotoxicity. However, prior exposure of NK cells to *Leishmania* parasites synergistically increased their IFN-γ release in response to IL-12, which was dependent on endogenous IL-18. CD1c^+^ dendritic cells were identified as possible source of *Leishmania*-induced IL-12. Finally, we observed that direct contact between *Leishmania* and NK cells reduced the expression of CD56 mRNA and protein on NK cells. We conclude that *Leishmania* activate NK cells *via* trans-presentation of IL-18 by monocytes and by a monocyte-derived soluble factor. IL-12 is needed to elicit the IFN-γ-response of NK cells, which is likely to be an important component of the innate control of the parasite.

## Introduction

Natural killer (NK) cells are members of the innate lymphoid cells (ILCs), which do not express rearranged antigen receptors and are characterized by an absent or only slow clonal expansion. Based on their ability to rapidly release the T helper (Th) 1 signature cytokine interferon (IFN)-γ upon stimulation, NK cells belong to the type 1 ILCs. However, in contrast to other ILC1s NK cells are developmentally dependent on eomesodermin (eomes), require interleukin (IL)-15 instead of IL-7 for cell survival, and kill virally infected or tumor cells by exocytosis of cytotoxic granules [reviewed in Ref. (1)].

As a first sign of activation mouse and human NK cells upregulate the expression of surface CD69, a type II C-type lectin absent from resting NK cells (2–5). CD69 is a costimulatory molecule which is able to enhance NK cell effector functions (6). Depending on the activation signal NK cells can also produce soluble mediators other than IFN-γ including pro- [e.g., tumor necrosis factor (TNF)] or anti-inflammatory cytokines (e.g., IL-10), growth factors (e.g. granulocyte-monocyte colony-stimulating factor), and chemokines (e.g., CCL2-5 and CXCL8) [reviewed in Ref. (7)]. Based on these properties, mouse and human NK cells exert various immunoregulatory functions and contribute not only to the antitumor response, but also to the defense against viruses, bacteria, fungi, and parasites (8–15).

To acquire full effector capacity NK cells require priming by cytokines and accessory cells such as dendritic cells (DCs) (16–19). Cytokines that activate human NK cells include IFN-α/β (20, 21), IL-1 (22), IL-2 (23), IL-12 (24), IL-15, IL-18 (25), IL-21 (26), and IL-27 (27). In most cases, a combination of at least two cytokines is needed to achieve a full NK cell response. In addition, NK cells can also be activated by ligation of pattern recognition receptors such as toll-like receptors (TLRs) or by differential engagement of activating and inhibitory NK cell receptors [reviewed in Ref. (7, 28)]. One of the NK activating receptors, NKp46 [*syn*. natural cytotoxicity triggering receptor (NCR)1] represents the most specific NK cell marker in mammalian organisms (29). Besides NKp46, CD56, also known as neural cell adhesion molecule 1 (NCAM1), is commonly used to define human NK cells as CD56^+^CD3^−^ cells. In humans, the two main NK cell effector functions, cytotoxicity and cytokine production, have been associated with two distinct NK cell subsets: CD56^bright^CD16^−^ NK cells that predominate in lymphatic tissues and are specialized in IFN-γ secretion, and CD56^dim^CD16^+^ NK cells that are mainly present in peripheral blood and show cytotoxic activity (30, 31). However, dependent on the mode of activation, both NK cell subpopulations may also exhibit the “non-specialized” NK cell effector function (32–34). The function of CD56 on NK cells is largely unknown, but published data indicate a relationship between the height of CD56 expression and the degree of activation (35).

*Leishmania* are protozoan parasites with a dimorphic cell cycle. The flagellated, promastigote form of *Leishmania* is transmitted by the bites of sand flies. In the mammalian host, the promastigotes are endocytosed by phagocytic cells and transform into the aflagellated stage (amastigotes) that replicates within phago(lyso)somes (36). Depending on the *Leishmania* species and strain and the immune response and genetic background of the host, infections can be asymptomatic, lead to self-healing or chronic cutaneous leishmaniasis (CL; e.g., *Leishmania major, Leishmania mexicana*) or non-healing, progressive mucocutaneous leishmaniasis (e.g., *Leishmania braziliensis*), or can cause visceral leishmaniasis (VL; *Leishmania infantum* and *Leishmania donovani*) due to systemic spreading of the parasites (37). Experimental animal models of CL and VL led to the identification of the key immune mechanisms required for the control of infection, which include the generation of IL-12 and TNF, the expansion of IFN-γ-producing CD4^+^ and CD8^+^ T cells and the induction of antileishmanial effector pathways such as inducible nitric oxide synthase (iNOS). By contrast, induction of macrophage-deactivating cytokines such as IL-10 and transforming growth factor (TGF) β as well as overshooting production of Th2 cytokines were associated with disease progression [reviewed in Ref. (38–40)]. Many of the above-mentioned mechanisms also hold true in human leishmaniasis, as biopsies of chronic CL lesions and leukocytes of VL patients displayed high IL-10 and TGFβ content, whereas cells of cured patients produced IL-12 and IFN-γ (41–46).

Natural killer cells were found to participate in the innate control of *Leishmania* in infected mice but were not essential for generating a Th1 response and ultimate healing of the disease [reviewed in Ref. (13)]. During later stages of VL, mouse NK cells showed adverse effects and inhibited protective immunity in an IL-10-dependent manner (47). The protective function of NK cells in murine leishmaniasis is largely due to their release of IFN-γ and subsequent stimulation of iNOS-dependent killing of parasites, as they were not able to recognize *Leishmania*-infected host cells as targets for direct cytolysis *in vitro* and *in vivo* (48). During the early phase of infection, NK cell activation in *Leishmania*-infected mice required DC- and TLR9-dependent production of IL-12, T cell-mediated release of IL-2, and the presence of IL-18 (18, 49, 50). In *L. major* infections of mice, IFN-α/β was necessary for full NK cell activation (51). *Leishmania* parasites failed to directly activate mouse NK cells (18).

Several observations argue for a protective role of NK cells also in human leishmaniasis. These include (a) a reduced NK cell number in the blood of patients with acute VL that was restored after successful chemotherapy; (b) the influx of NK cells into lesions of CL patients, who showed suppressed NK cell cytotoxicity during active disease, but positive response to treatment (52–54); and (c) a reduced number, TLR expression and IFN-γ and TNF-production by NK cells in patients with diffuse as compared with localized CL due to *L. mexicana* infection (55, 56). Unlike to murine NK cells, mechanisms of human NK cell activation are less clear. Some studies claimed indirect stimulation of human blood NK cells by accessory cells releasing cytokines after contact to *Leishmania* (57–59). Other reports suggested direct activation of NK cells by *Leishmania* in a lipophosphoglycan (LPG)/TLR2-dependent or LPG-independent manner (60, 61) or even excluded a NK cell IFN-γ response in *Leishmania*- or *Leishmania* antigen-stimulated peripheral blood mononuclear cells (PBMCs) (62, 63).

To define the activation signals required for a human NK cell effector response to *Leishmania* parasites and to address the question whether there are differences between *Leishmania* species, we performed cocultures of human PBMCs or highly purified cell populations from healthy German volunteers with four different *Leishmania* species and analyzed the NK cell response. The data obtained show that NK cells cannot be directly activated by *Leishmania* promastigotes but require cytokine signals from monocytes.

## Materials and Methods

### *Leishmania* Parasites

Promastigotes of the following *Leishmania* species and strains were used: *L. infantum* MHOM/DE/98/LUB1 [isolated in our laboratory from bone marrow (BM) of a German patient with VL] (64), *L. infantum* MHOM/DE/2012/VA21737 (isolated in our laboratory from BM of a German patient with VL), *L. infantum* MHOM/DE/2014/VA20763 (isolated in our laboratory from the skin lesion of a Croatian patient with CL), *L. infantum* MCAN/ES/2010/BON (isolated in our laboratory from peripheral blood of a Swiss dog with VL), *L. major* MHOM/IL/1981/FEBNI (isolated from the skin lesion of an Israeli patient with CL) (65), *L. mexicana* MNYC/BZ/1962/M379 [isolated from a vesper rat (ATCC^®^ 50156™); kindly provided by Sigrid Roberts, Hillsboro, OR, USA] and *L. donovani* (MHOM/SD/1962/1S-CL2D clonal line LdBob; originally isolated from a Sudanese patient with VL; kindly provided by Steve Beverley, St. Louis, MO, USA) (66). In case of *L. infantum*, the strain MHOM/DE/98/LUB1 was used unless otherwise stated. For all strains, aliquots of a promastigote culture (derived from amastigotes isolated from experimentally infected mice) were frozen after only two to three *in vitro* passages for expansion. All experiments were performed with freshly thawed aliquots of these promastigotes which were grown at 28°C/5% CO_2_/95% humidified air in modified Schneider’s *Drosophila* insect medium as described (67) for a maximum of six *in vitro* passages. For fixation of promastigotes, parasites were incubated for 10 min in 4% paraformaldehyde (Pfa) at room temperature (RT) followed by three washes with PBS. Freeze–thaw (ft) lysates of *Leishmania* promastigotes were generated by four cycles of freezing at −80°C and thawing at RT.

### PBMC Preparation and Purification of Different Cell Populations from the Blood

Mononuclear cells from EDTA-anticoagulated human peripheral blood (PBMCs) of healthy human volunteers living in Erlangen and without any history of leishmaniasis were isolated using density centrifugation (1.077 g/ml Biocoll, Biochrom). For generation of autologous plasma, blood was first centrifuged, and the resulting plasma supernatant (SN) was heat inactivated (56°C, 30 min) and filtered, while the remaining cell pellet was resuspended in PBS to proceed with PBMC preparation. Different cell populations of PBMCs including CD3^+^CD56^−^ T cells, CD19^+^ B cells, CD56^+^CD3^−^ NK cells, CD14^+^ monocytes, CD14^high^CD16^−^CCR2^high^ classical monocytes, CD14^low^CD16^high^CCR2^−^ non-classical monocytes, and CD14^high^CD16^low^CCR2^+^ intermediate monocytes were purified by cell sorting using the FACS Aria II instrument (BD Biosciences). Purification of CD1c^+^ DCs was performed as described with slight modifications (68). Briefly, leukocyte reduction cones retrieved from anonymous healthy adult donors were used as source for PBMCs. CD1c^+^ DCs were then enriched with the EasySep Pan-DC Pre-Enrichment Kit (Stemcell Technologies) and isolated by cell sorting using an FACS Aria II (BD Bioscience) as CD3^−^CD14^−^CD19^−^CD20^−^CD56^−^HLA-DR^+^CD1c^+^CD11c^+^ cells. All sorted cell populations showed a purity of >96%. For immunofluorescence analysis, monocytes were purified by negative selection using the Monocyte Isolation Kit II (Miltenyi Biotech) following the manufacturer’s instructions.

### Culture and Stimulation of Cells

Human leukocytes with or without *Leishmania* promastigotes were cultured in 96-well plates (PBMCs: 5 × 10^5^ cells/well, 200 µl; purified NK cells: 0.5–1.5 × 10^5^/well depending on the total recovery, 200 µl), 48-well plates (PBMCs: 10^6^ cells/well, total volume 500 µl; purified monocytes: 5 × 10^5^/well, 500 µl), or in 24-well plates with a transwell (TW) insert (0.4 µm pore size; Corning, Wiesbaden, Germany; 5 × 10^5^ NK cells/insert, 0.5–1 × 10^6^ monocytes/bottom well) at 37°C and 5% CO_2_/95% humidified air for 20 h using RPMI1640 (Gibco™ Life Technologies; ThermoFisher Scientific, cat. no. 21875-034) supplemented with 10 mM HEPES (ThermoFisher Scientific), 50 µM 2-mercaptoethanol (Sigma-Aldrich), 100 U/ml penicillin and 100 µg/ml streptomycin (ThermoFisher Scientific), and 10% heat-inactivated autologous plasma. *Leishmania* promastigotes were added at different parasite/host cell ratios [multiplicity of infection (MOI) 0.2, 1, 5, 10, or 33]. Pfa-fixed *Leishmania* promastigotes or *Leishmania* freeze–thaw lysate were used in analogy to the MOI of viable parasites. When different purified leukocyte populations (e.g., NK cell and monocytes) were cocultured with *Leishmania* promastigotes, cell populations were used at the same ratios as present in non-separated PBMCs of this donor, unless otherwise stated. In some of the experiments, leukocyte/promastigote cocultures were incubated in the presence of specific blocking antibodies (Abs) against different cytokines [sheep-anti-IFN-α (1:350; 10,000 neutralizing units/ml) or sheep-anti-IFN-β antiserum (1:3; 1,000 neutralizing units/ml), obtained from the NIAID Repository, Braton Biotech Inc., Rockville, MD, USA; mouse-anti-IL-1β, 10 µg/ml, CRM56, eBioscience/ThermoFisher Scientific; mouse-anti-IL-2, 1 µg/ml, AB12-3G4, eBioscience/ThermoFisher Scientific; rat-anti-IL-6, 5 µg/ml, MQ2-13A5, BioLegend; mouse-anti-IL-12/IL-23p40, 20 µg/ml, C11.5, BioLegend; mouse-anti-IL-15, 1 µg/ml, ct2n, eBioscience/ThermoFisher Scientific; and mouse-anti-IL-18, 1.5 µg/ml, 125-2H, MBL] or the respective control sera or isotype control Abs. To verify the efficacy of the Ab-treatment, cells were stimulated with the appropriate recombinant cytokine. Cytokines/chemokines used were as follows: huIFN-α and huIFN-β (100 U/ml; NIAID Repository, Braton Biotech Inc., Rockville, MD, USA), rhuIL-1β (20 ng/ml, PeproTech), rhuIL-2 (200 U/ml; Chiron, Emeryville, CA, USA), rhuIL-4 (250 U/ml, PeproTech), rhuIL-6 (10 ng/ml, BioLegend), rhuIL-8 (10 ng/ml, BioLegend), rhuIL-12p70 (10 ng/ml, PeproTech), rhuIL-15 (12 ng/ml, PeproTech), rhuIL-18 (10 ng/ml, MBL), and rhuMIP1α (20 ng/ml, BioLegend). In addition, PBMCs or purified NK cells were stimulated with cell culture SNs (vol/vol 20–80%) of previous leukocyte/*Leishmania* cocultures of the same donor. In some cases, the SNs were incubated with blocking Abs to cytokines (see above) for 1–2 h at 37°C and 5% CO_2_/95% humidified air before being added to freshly isolated cells.

As a positive control for the stimulation of DCs resiquimod (R848, 5 µg/ml, InvivoGen), a TLR7/8 agonist, was used.

### Cytokine Measurements in Cell Culture SNs

Commercial ELISA kits were used for determining the content of human IL-2, IL-6, IL-18, or IL-12p35/70 (eBioscience/ThermoFisher Scientific) and IL-10 or IL-12/IL-23p40 (BioLegend) in culture SNs. Multiplex ELISA was performed using the ProcartaPlex^®^ Multiplex Immunoassay (Human Cytokine/Chemokine/Growth Factor Panel 1, 45 plex; eBioscience/ThermoFisher Scientific), which was analyzed with a MAGPIX^®^ instrument and the xPONENT^®^ software (eBioscience/ThermoFisher Scientific).

### Flow Cytometry

For surface phenotyping and cell sorting of freshly isolated or cultured leukocytes, fluorochrome-labeled or biotinylated Abs against the following antigens were used (all from eBioscience/ThermoFisher Scientific, unless otherwise stated): CD3ε (OKT3, FITC, PerCP-Cy5.5, biotinylated), CD11b (ICRF44, V450, BD Biosciences), CD11c (3.9, PerCP-eflour^®^710), CD14 (61D3, FITC), CD16 (CB16, PerCP-eflour^®^710, eflour^®^450), CD19 (HIB19, PE, eflour^®^450), CD25 (BC96, PE-Cy7), CD56 (CMSSB, PE-Cy7, APC), CD69 (FN50, PerCP-Cy5.5, BioLegend), CD192/CCR2 (K036C2, PerCP-Cy5.5, PE-Cy7; BioLegend), and CD335/Nkp46 (9E2, PE, Miltenyi Biotech). Staining with biotinylated Abs was followed by incubation with fluorochrome (FITC or APC)-labeled streptavidin (BD Biosciences) to allow detection.

Staining of blood CD1c^+^ DCs was done as described with minor modifications (68). Briefly, after enrichment of human DCs with the EasySep Pan-DC Pre-Enrichment Kit (Stemcell Technologies), cells were stained with fluorochrome-coupled Abs against CD1c (L161, APC/Cy7, BioLegend), CD3 (UCHT1, BUV395, BD Bioscience), CD11b (M1/70, Alexa Fluor 700, BioLegend), CD11c (3.9, PE/Cy7, BioLegend), CD14 (HCD14, Alexa Fluor 700, BioLegend), CD19 (HIB19, V450, BD Bioscience), CD20 (2H7, eflour^®^450), CD56 (5.1H11, Brilliant Violet 421, BioLegend), CD123 (6H6, BV605, BioLegend), CD141 (1A4, Brilliant Violet 711, BD Bioscience), CD303a (201A, PerCP-Cy5.5, BioLegend), and HLA-DR (L243, Brilliant Violet 510, BioLegend) for 30 min on ice.

The specificity of the stainings was verified by use of isotype control Abs. Cells were analyzed with an FACS Canto II system and Diva 6.1.2 (both BD Biosciences) and FlowJo 10.0.7 (FlowJo LLC, Ashland, OR, USA) software. DCs were analyzed with an FACS LSRFortessa™. For intracellular staining of IFN-γ, GolgiStop™ (1:1,500 µg/ml, BD Biosciences) was added during the final 6–10 h of cell culture to prevent secretion of cytokines. After staining of surface molecules, cells were fixed by Cytofix/Cytoperm™ (BD Biosciences), washed twice with a saponin-containing buffer, and stained for intracellular accumulated IFN-γ (α-huIFN-γ, 4S.B3, APC) (18).

### Confocal Laser Scanning Fluorescence Microscopy (CLSFM) of Infected Monocytes

After coculture of untouched purified monocytes with *Leishmania* promastigotes (MOI 10) for 20 h, 2.5 × 10^5^ cells in 30 µl were transferred to the marked reaction field of adhesion slides (Marienfeld Laboratory Glassware) prepared as recommended by the manufacturer. After cell adhesion, slides were washed twice in PBS buffer and cells were fixed with 4% Pfa. Fixed monocytes were either directly stained or additionally permeabilized with methanol (−20°C) before staining. For IL-18 staining, non-specific binding sites were blocked with PBS/2% BSA/10% normal goat serum and cells were stained with mouse-anti-IL-18 monoclonal Ab (125-2H, MBL) overnight at 4°C. As specificity control, the mouse-anti-IL-18 mAb was pretreated with rhuIL-18 (1.2 µg/ml, 30 min, 37°C). All Abs were diluted in PBS/0.5% BSA/0.5% normal goat serum. After washing with PBS/0.1% Tween Alexa Fluor 568-conjugated goat anti-mouse secondary Abs (ThermoFisher Scientific) were added for 30 min at RT. Cell nuclei were visualized by DAPI staining. Slides were mounted in Vectashield (Vector laboratories) and cover slips, dried in the dark for at least 12 h at 4°C, and analyzed by CLSFM (LSM700, Zeiss) using a 63× objective. Image processing was performed using the ZEN software 2009 (Zeiss).

### Cytotoxicity Assay

Peripheral blood mononuclear cells (with the percentage of Nkp46^+^CD3^−^ NK cells determined by flow cytometry) were added to K562 tumor target cells in NK cell/target cell ratios of 20:1, 10:1, 5:1, and 2.5:1. A standard chromium-51 release assay was performed (48). Briefly, K562 tumor cells were labeled with ~150 μCi ^51^Cr (Perkin-Elmer) for 90 min. Cocultures of effector and target cells were incubated in complete RPMI1640 medium containing 10% heat-inactivated fetal calf serum (Sigma-Aldrich, cat. no. F-7524, lot. no. 036K3397) for 4 h. The release of ^51^Cr into the SNs was measured as counts per minute (cpm) using a TopCount NXT microplate gamma counter (Perkin-Elmer). Based on the spontaneous (target cells alone) and the maximum release (^51^Cr-labeled cells directly added to the LUMA measurement plate) % specific lysis was calculated as (cpm_sample_ − cpm_spontaneous_)/(cpm_maximum_ − cpm_spontaneous_) × 100.

### RNA Preparation and Quantitative RT-PCR

Total RNA was prepared with the RNeasy Mini Kit (Qiagen). cDNA synthesis and quantitative RT-PCR analysis were performed (49) using the following assays: NCAM1 (CD56) (Hs00941830_m1), GAPDH (Hs02758991_g1).

### Statistical Analysis

Results were displayed as mean ± SEM or as median and were statistically analyzed by the Mann–Whitney *U* test using GraphPad Prism software v.4. Significant differences between unstimulated and stimulated samples were marked by asterisks, significant differences between stimulated samples by diamonds. Significant *p* values are indicated as follows: *^,#^*p* < 0.05; **^,##^*p* < 0.01; ***^,###^*p* < 0.001.

## Results

### NK Cells within PBMCs, but Not NK Cells Alone Upregulate CD69 in Response to *Leishmania*

**Figure 1:**
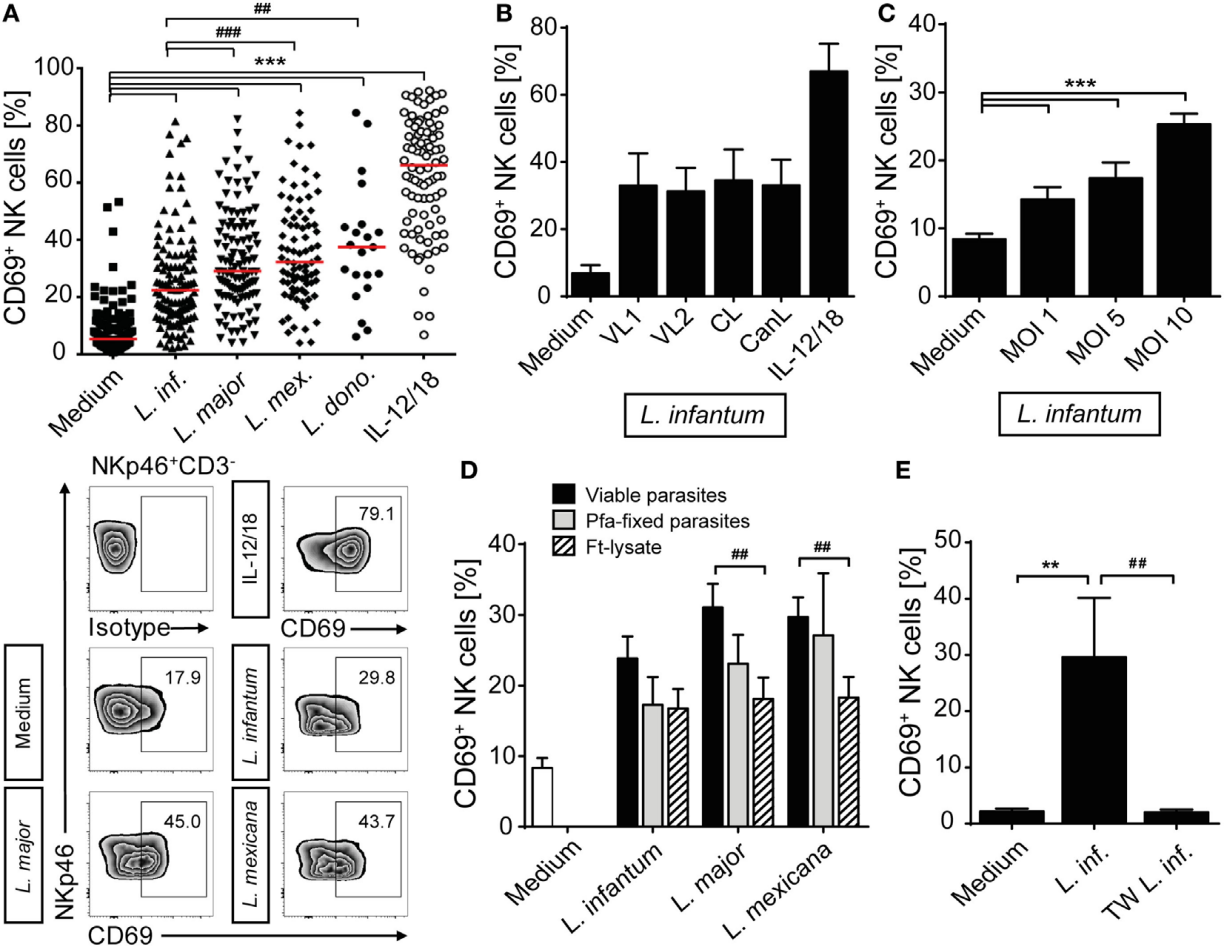
CD69 is upregulated on natural killer (NK) cells after contact with *Leishmania*. Human peripheral blood mononuclear cells were cocultured for 20 h with *Leishmania* promastigotes of different species **(A)**, strains **(B)**, amounts **(C)**, or integrity **(D)**, and with or without host cell/parasite contact **(E)** or interleukin (IL)-12 and IL-18 (10 ng/ml) **(A,B)**, followed by surface expression analysis of CD69 on NKp46^+^CD3^−^ NK cells by flow cytometry. Unless otherwise indicated the multiplicity of infection (MOI) was 10. **(A)** Results of 129/117/107/83/21/88 blood samples for the six stimulations. Medians are indicated by red lines. FACS plots show results of NKp46^+^CD3^−^ NK cells of one representative experiment. **(B)** *Leishmania infantum* strains isolated from human patients with visceral leishmaniasis (VL) (VL1, VL2) or CL or a dog [canine leishmaniasis (CanL)]. Mean ± SEM of 9/9/9/9/7/9 donors. **(C)** Mean ± SEM of 119/43/17/119 donors for the four stimulations. **(D)** Mean ± SEM of 22/22/18/18 (viable), 8/9/6 [paraformaldehyde (Pfa)-fixed], and 17/15/14 [freeze–thaw lysate (Ft-lysate)] donors. **(E)** Mean ± SEM of six donors. Abbreviation: TW, transwell. *^,#^*p* < 0.05; **^,##^*p* < 0.01; and ***^,###^*p* < 0.001 two-tailed Mann–Whitney *U* test.

To investigate whether *Leishmania* parasites themselves and/or host cell-derived factors activate human NK cells, PBMCs of healthy volunteers without history of leishmaniasis were cultured in the presence of promastigotes of different *Leishmania* species. Blood NK cells gated as NKp46^+^CD3^−^ viable single cells were analyzed for surface expression of the early activation marker CD69 by flow cytometry after 6, 12, and 20 h of incubation. As induction of CD69 was most prominent after 20 h (Figure S1 in Supplementary Material) and the viability of NK cells decreased thereafter, this time point was chosen for further analyses. Stimulation with promastigotes of all *Leishmania* species tested induced upregulation of CD69 on human NK cells in a large number of different blood donors (total of 36), most of which were tested several times in independent experiments (Figure 1A). The average induction of CD69^+^ NK cells by *L. infantum* was lower than by *L. major, L. mexicana*, and *L. donovani*. There were no differences observed between viscerotropic and dermotropic human strains or a canine strain of *L. infantum* (Figure 1B). The percentage of CD69^+^ NK cells increased with the parasite/host cell ratio used (Figure 1C). Fixed or lysed parasites still caused an induction of CD69 on NK cells, which, however, was tentatively or significantly reduced when compared with viable parasites (Figure 1D). By contrast, CD69 induction on NK cells was abolished when *Leishmania* promastigotes were separated from PBMCs by a membrane (pore size 0.4 µm) (Figure 1E).

**Figure 2:**
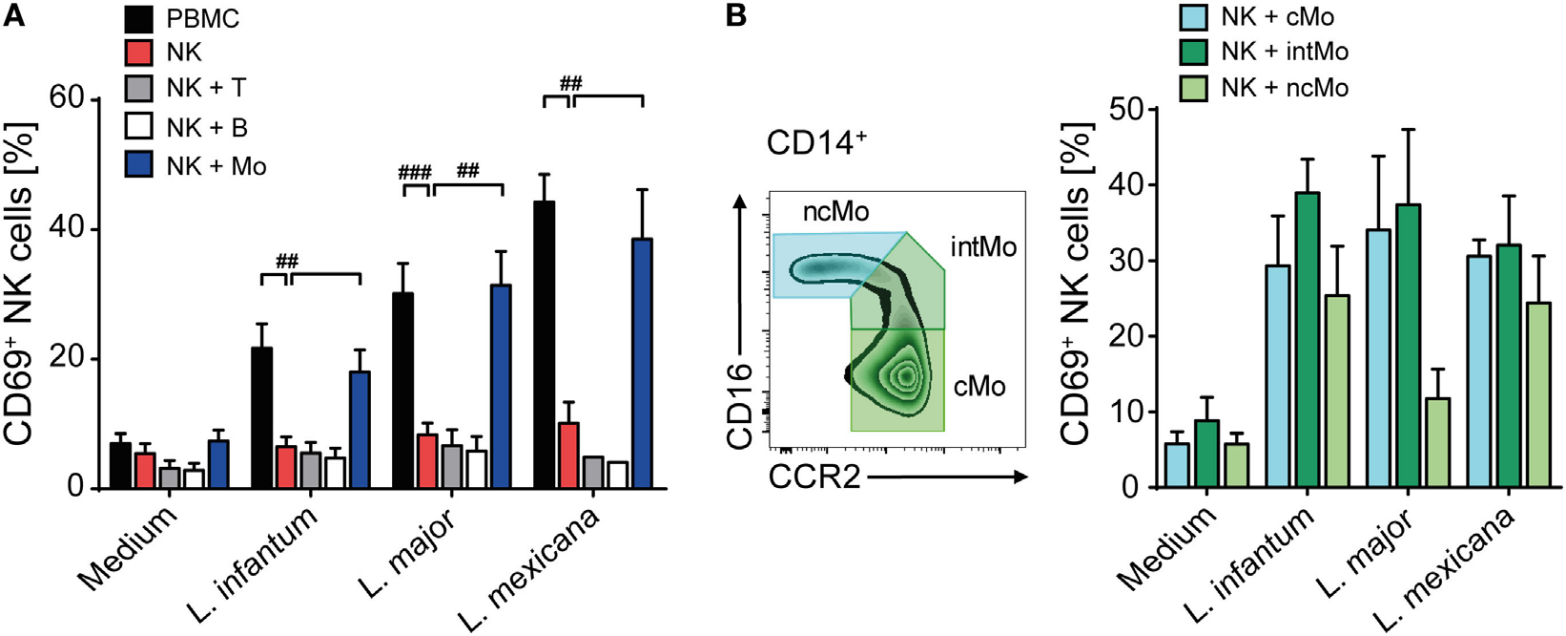
Upregulation of CD69 on natural killer (NK) cells after coculture with *Leishmania* requires the presence of monocytes. **(A)** Peripheral blood mononuclear cells (PBMCs) or sorted NKp46^+^CD3^−^ NK cells with or without autologous, sorted CD3^+^NKp46^−^ T cells, CD19^+^ B cells, or CD14^+^ monocytes were cocultured with *Leishmania* spp. promastigotes (multiplicity of infection 10). **(B)** Using the surface markers CD16 and CCR2, classical, intermediate, or non-classical CD14^+^ monocytes were sorted and cocultured with autologous sorted NK cells and *Leishmania* spp. promastigotes (MOI 33). After 20 h, surface expression of CD69 on NKp46^+^CD3^−^ NK cells was determined by flow cytometry. Mean ± SEM of **(A)** 15/14/14/7 (PBMC/NK + Mo), 15/14/14/4 (NK), and 7/7/6/2 (NK + T/NK + B) and **(B)** 5/5/4/4 donors for the four stimulations. ^#^*p* < 0.05, ^##^*p* < 0.01, and ^###^*p* < 0.001 two-tailed Mann–Whitney *U* test.

Having seen that direct contact between *Leishmania* and NK cells or other cell types within the PBMC culture was necessary to upregulate CD69, we next investigated whether the parasite was able to directly activate human NK cells. To this end, highly purified CD3^−^NKp46^+^ NK cells as well as whole PBMCs of the very same donor were stimulated by promastigotes (Figure 2A). Whereas NK cells within the PBMC/*Leishmania* coculture readily upregulated CD69, purified NK cells failed to do so, irrespective of the *Leishmania* species used. From these data we conclude that the upregulation of CD69 on NK cells in response to *Leishmania* is dependent on the presence of accessory cells.

### Upregulation of CD69 on NK Cells Requires Infected Monocytes Trans-Presenting IL-18

To elucidate which additional cell population is needed to activate human NK cells in response to promastigotes, CD3^+^Nkp46^−^ T cells, CD19^+^ B cells, and CD14^+^ monocytes were sorted and added separately to a coculture of *Leishmania* and purified NK cells of the same donor. Addition of monocytes to the NK cell/parasite culture restored induction of CD69 on NK cells to a similar level as observed in whole PBMC cocultures, whereas addition of T or B cells did not support the expression of CD69 on NK cells (Figure 2A). As human CD14^+^ monocytes are subdivided in classical (cMo, CD14^high^CD16^−^CCR2^high^), intermediate (intMo, CD14^high^CD16^low^CCR2^+^), and non-classical monocytes (ncMo, CD14^low^CD16^high^CCR2^−^) (69), the three subpopulations were purified and evaluated for their capacity to induce CD69 on NK cells after a 20 h coculture with *Leishmania* and purified NK cells. All three types of monocytes were able to induce CD69 on NK cells in response to *Leishmania* (Figure 2B).

**Figure 3:**
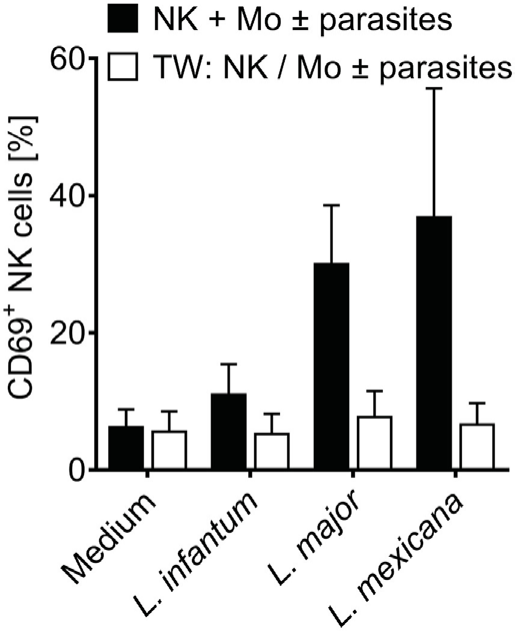
CD69 induction on natural killer (NK) cells requires contact with infected monocytes. Sorted NKp46^+^CD3^−^ NK cells and autologous sorted CD14^+^ monocytes were cocultured in the presence of *Leishmania* spp. promastigotes (multiplicity of infection 10 relative to the number of NK cells), either in one single well or in a transwell (TW) system in which the NK cells (in the insert) were separated from the monocytes and *Leishmania* (in the bottom well) by a membrane (pore size 0.4 µm). After 20 h, CD69 surface expression of NKp46^+^CD3^−^ NK cells was determined by flow cytometry. Mean ± SEM of 6/5/6/3 and 3/5/6/3 donors for the four stimulations.

To define whether infected monocytes stimulated NK cells *via* a soluble factor or by a cell contact-dependent mechanism, a TW system was used. Whereas NK cells of a mixed NK cell/monocyte/*Leishmania* culture showed an increase in CD69 expression after 20 h of incubation, NK cells that were separated from infected monocytes by a membrane did not (Figure 3). Likewise, NK cells did not become activated in TW experiments, in which NK cells and *Leishmania* were separated from uninfected monocytes (two independent experiments, data not shown). Thus, direct contact between infected human monocytes and NK cells is essential to upregulate CD69 on NK cells.

**Figure 4:**
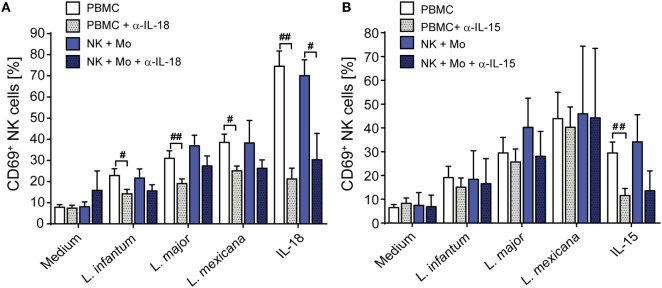
Upregulation of CD69 on natural killer (NK) cells depends on interleukin (IL)-18. Peripheral blood mononuclear cells (PBMCs) or sorted NKp46^+^CD3^−^ NK cells ± autologous, sorted CD14^+^ monocytes were cocultured with *Leishmania* spp. promastigotes (multiplicity of infection 10) in the presence or absence of neutralizing antibodies against **(A)** IL-18 (1.5 µg/ml) or **(B)** IL-15 (1 µg/ml). After 20 h, the CD69 surface expression of NKp46^+^CD3^−^ NK cells was determined by flow cytometry. Mean ± SEM of **(A)** 16/15/16/8/5 (PBMCs) or 8/8/8/5/6 (NK + Mo) donors and **(B)** 19/8/6/3/10 (PBMCs) or 2/2/2/2/2 (NK + Mo) donors for the five different stimulation conditions. ^#^*p* < 0.05, ^##^*p* < 0.01, and ^###^*p* < 0.001 two-tailed Mann–Whitney *U* test.

**Figure 5:**
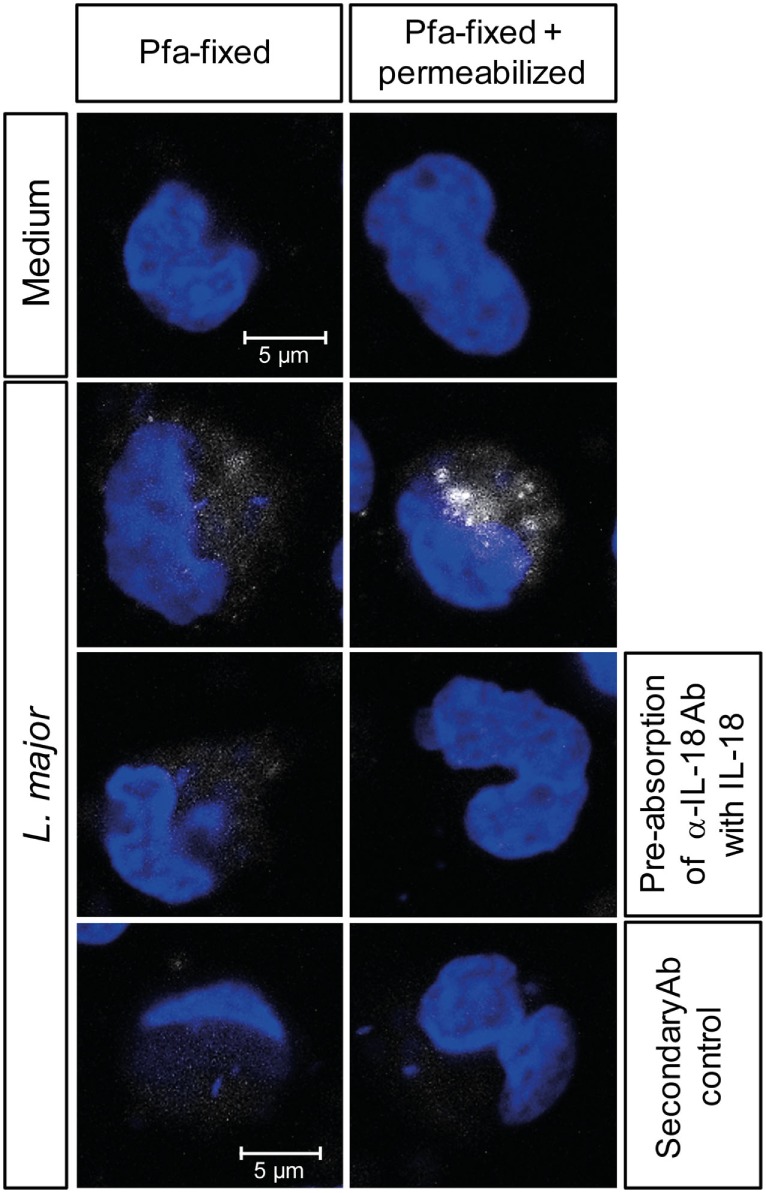
Interleukin (IL)-18 is detectable on fixed, but non-permeabilized monocytes after exposure to *Leishmania*. Purified CD14^+^ monocytes attached to an adhesion slide were incubated with or without *Leishmania major* promastigotes (multiplicity of infection 10) for 20 h. Thereafter, monocytes were either fixed with paraformaldehyde (Pfa) or fixed with Pfa and permeabilized with methanol, before being stained for IL-18 (white) and with DAPI (blue). As controls, the mouse-anti-IL-18 antibody (Ab) was pre-absorbed with rhuL-18, or the cells were incubated with the secondary Ab alone. Representative images of one of three independent experiments are shown.

In murine leishmaniasis NK cell activation is mediated by cytokines [reviewed in Ref. (13)]. We therefore hypothesized that this might also apply for human NK cells and screened for NK cell-activating cytokines that are trans-presented by myeloid cells to the respective receptor on NK cells without being necessarily secreted. As both IL-15 and IL-18 were reported to be trans-presented by human monocytes (70, 71), we tested whether they are involved in the induction of CD69. Using neutralizing Abs, we found that the expression of CD69 on NK cells in infected PBMCs was partially dependent on IL-18 (Figure 4A). In the case of NK cell/monocyte/*Leishmania* cocultures, a similar effect was observed, which, however, did not quite reach the level of significance (Figure 4A). By contrast, neutralization of IL-15 did not affect the expression of CD69 on NK cells (Figure 4B). Two observations argue for monocyte-mediated trans-presentation rather than secretion of IL-18: first, in most of the SNs of PBMC/*Leishmania*, NK cell/monocyte/*Leishmania* or monocyte/*Leishmania* cocultures IL-18 was not measurable by ELISA (detection limit was 20 pg/ml); only in few of them (mostly after *L. major* stimulation) low levels of IL-18 (≤500 pg/ml) were found (Table S1 in Supplementary Material). Second, IL-18 was visualized on the surface of purified monocytes which had been in contact with *L. major* for 20 h and were stained for IL-18 after fixation with Pfa without permeabilization (Figure 5). Permeabilization of infected monocytes intensified the IL-18 staining, because intracellular IL-18 became additionally detectable (Figure 5). Together, these data suggest that *Leishmania*-infected monocytes trans-present IL-18 to human NK cells.

### A Heat-Labile Soluble Factor of Monocyte/*Leishmania* Cocultures Contributes to the Upregulation of CD69 on Human NK Cells

**Figure 6:**
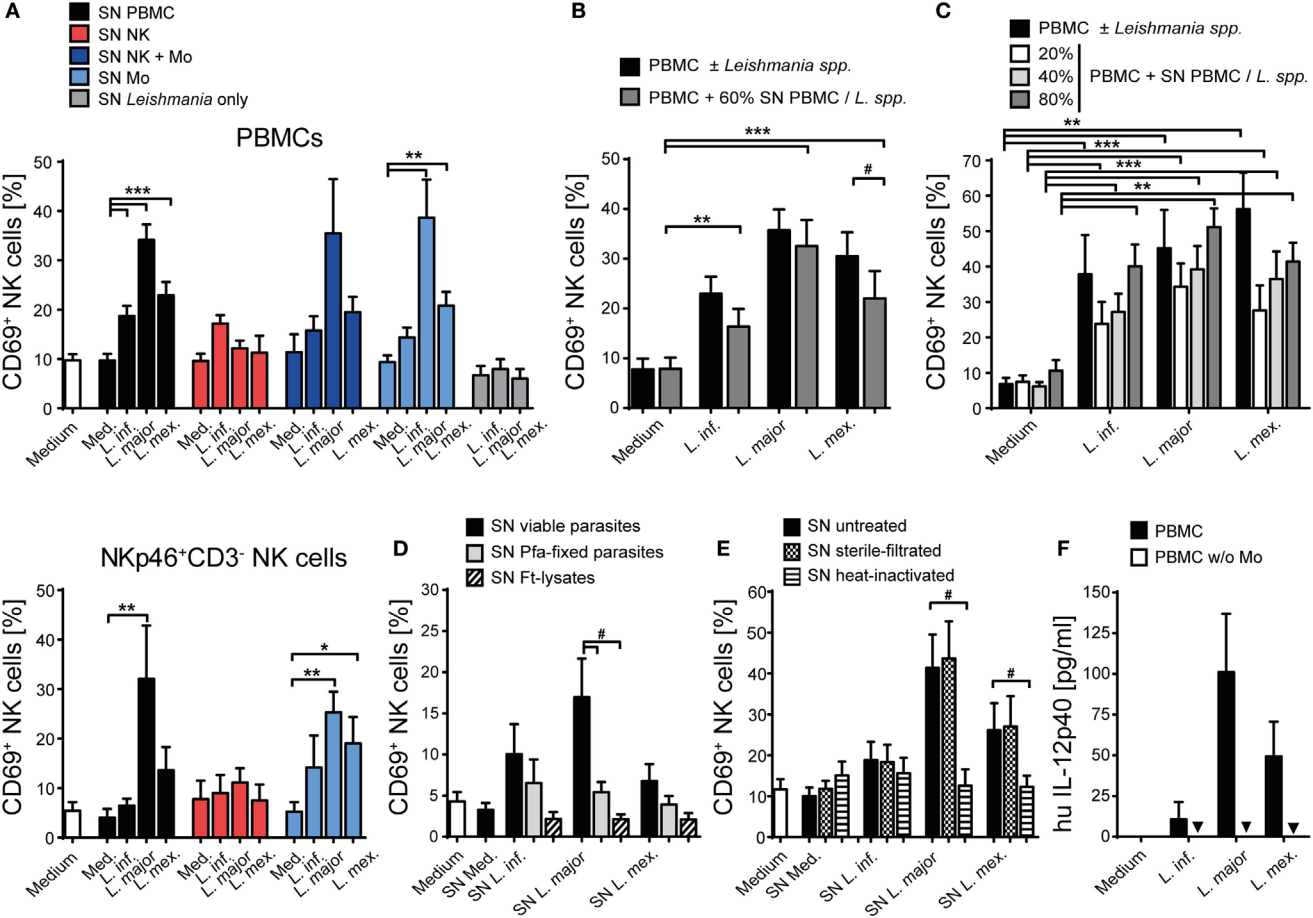
CD69 on natural killer (NK) cells is induced by a heat-labile soluble factor derived from infected monocytes. Human peripheral blood mononuclear cells (PBMCs) [**(A)** (top panel), **(B–E)**] or purified NKp46^+^CD3^−^ NK cells [**(A)** (bottom panel)] were stimulated with supernatants (SN) of various cell-*Leishmania*-cocultures for 20 h. CD69 expression of NKp46^+^CD3^−^ NK cells was determined by flow cytometry. Unless otherwise indicated **(C)**, SNs were added **(A,B,D,E)** at a final concentration of 60% (vol/vol) and used untreated **(A–D)**, sterile filtered **(E)**, or heat-inactivated **(E)**. [**(A)** top panel] Mean ± SEM of 59/59/50/49 (SN PBMC), 4/3/4/3 (SN NK), 4/4/4/2 (SN NK + Mo), 4/4/4/4 (SN Mo), or 8/8/6 donors (SN *Leishmania* only). [**(A)** bottom panel] Mean ± SEM of 7/7/6/5 (SN PBMC), 3/3/2/3 (SN NK), or 6/6/6/6 (SN Mo) donors for the four stimulations. **(B)** Mean ± SEM of 24/23/20/15 donors for the four stimulations. **(C)** Mean ± SEM of 11/4/3/4 (*Leishmania* spp.), 9/9/7/8 (20% SN), 10/11/7/7 (40% SN), or 9/9/7/9 (80% SN) donors for the four stimulations. **(D)** Mean ± SEM of seven (SN viable), seven (SN pfa-fixed), and four [SN freeze–thaw lysate (ft-lysate)] donors. **(E)** Mean ± SEM of 10/10/8/10 (SN untreated), 9/9/7/9 (SN filtered), and 9/9/6/8 (SN heat-inactivated) donors for the four stimulations. **(F)** PBMCs or PBMCs depleted of monocytes were cocultured with *Leishmania* spp. promastigotes (multiplicity of infection 10) for 20 h. The concentration of interleukin (IL)-12p40 in culture SNs was determined by ELISA (values below detection limit are marked by triangles). Mean ± SEM of 6/6/5/5 (PBMC) and 6/5/5/6 (PBMC w/o monocytes) donors for the four stimulations. *,#*p* < 0.05, **p < 0.01, and ***p < 0.001 two-tailed Mann–Whitney *U* test.

To investigate whether soluble factors released by infected monocytes are additionally involved in human NK cell activation, freshly isolated PBMCs or purified NK cells were incubated with culture SNs of *Leishmania*-stimulated (a) PBMCs, (b) purified NK cells (with or without monocytes), or (c) purified monocytes, all from the same blood donor. SNs of host cell-free *Leishmania* cultures were included as control. In the presence of SNs from previous PBMC/*Leishmania* or monocyte/*Leishmania* cultures, both NK cells within PBMCs and purified NK cells showed an upregulation of CD69, indicating that monocytes and/or *Leishmania* release a soluble factor after contact to each other that activates NK cells; SNs of promastigotes cultured without host cells had no effect (Figure 6A, upper and lower panels). The stimulatory effect of SNs derived from PBMC/*Leishmania* cocultures was concentration-dependent and, except for *L. mexicana*, as strong as direct stimulation of PBMCs by the parasite (Figures 6B,C). SNs of PBMCs incubated with dead parasites did not upregulate CD69 on NK cells (Figure 6D). The activity of the SNs was also lost, when they were boiled before addition to the PBMCs. By contrast, filtration (pore size 0.22 µm) did not influence their activity excluding host cell or parasite debris as stimuli (Figure 6E). Together, these data indicate that the NK cell stimulating activity of the SNs presumably results from a heat-labile protein released by infected monocytes.

**Figure 7:**
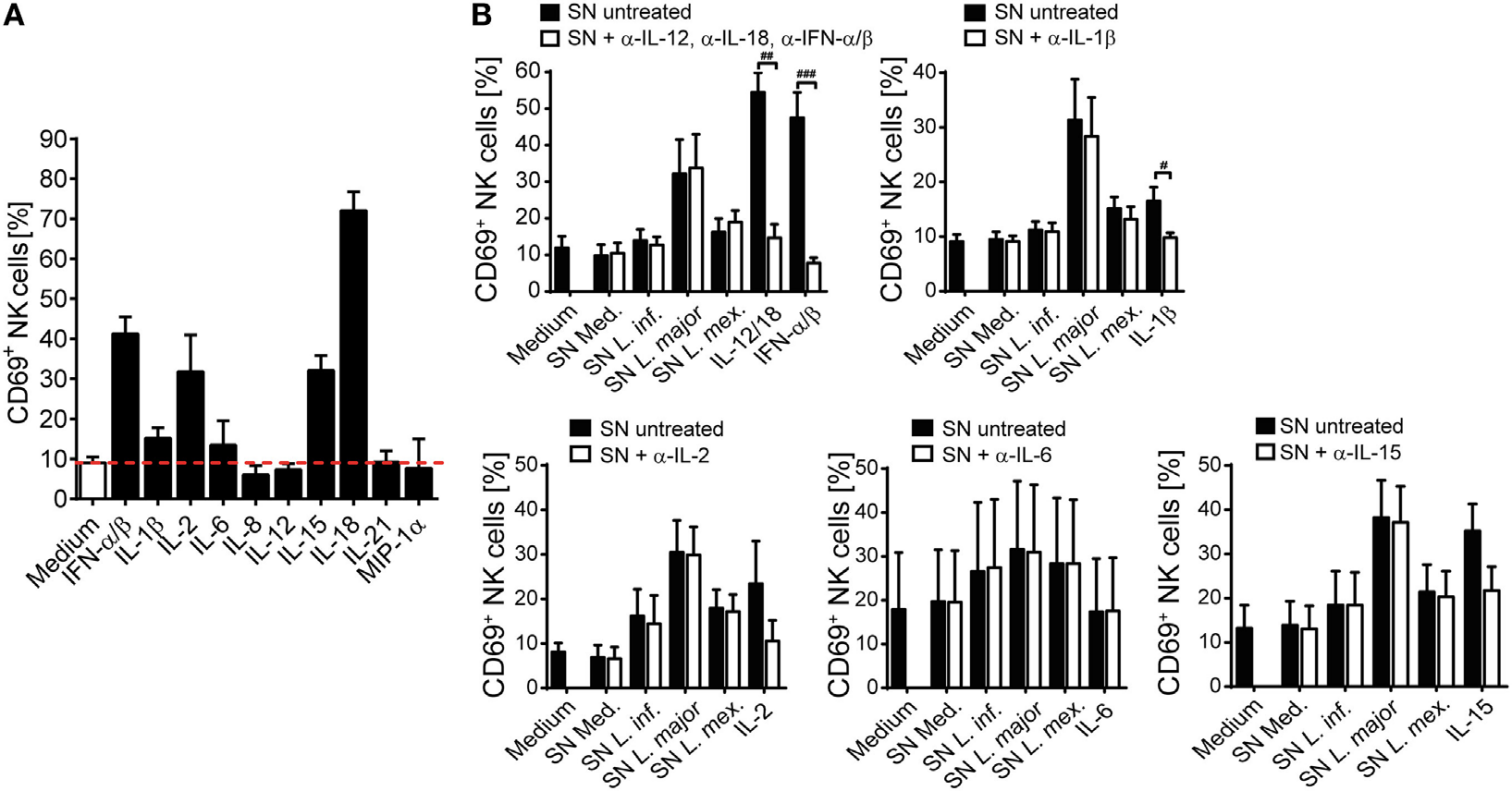
CD69 upregulation on natural killer (NK) cells by supernatants (SNs) of peripheral blood mononuclear cell (PBMC)/*Leishmania* cocultures is maintained after neutralization of NK cell-activating cytokines. Human PBMCs were stimulated with different **(A)** recombinant cytokines or **(A,B)** SNs of PBMC/*Leishmania* cocultures of the respective donors for 20 h, followed by analysis of CD69 surface expression on NKp46^+^CD3^−^ NK cells by flow cytometry. **(B)** Before stimulation, the cytokines or SNs were pretreated with either one or several neutralizing antibodies (Abs) (37°C, 1–2 h). The concentrations of the cytokines and Abs were as described in Section “Materials and Methods.” **(A)** Mean ± SEM of 57/57/17/11/12/8/19/8/7/3/3 donors for the 11 stimulations. **(B)** Mean ± SEM of 7/7/7/6/7/7/7 [SN + anti-interferon (IFN)-α/β, anti-interleukin (IL)-12, and anti-IL-18], 7/7/7/7/6/7 (SN + anti-IL-1β), 9/9/9/7/8/9 (SN + anti-IL-15), 5/5/5/5/5/5 (SN + anti-IL-2), or 4/4/4/4/4/4 (SN + anti-IL-6) donors for the different stimulations. ^#^*p* < 0.05, ^##^*p* < 0.01, and ^###^*p* < 0.001 two-tailed Mann–Whitney *U* test.

Next, we analyzed the spectrum of cytokines and chemokines secreted by *Leishmania*-activated monocyte/NK cell cultures of three different donors. We focused on *L. major*, because SNs from *L. major*-stimulated cell cultures were on average most potent in upregulating CD69 on NK cells. Using a Procarta^®^ Multiplex Immunoassay, substantial amounts of several cytokines and chemokines were measured in the SNs of all three tested individuals (Table S2 in Supplementary Material). In further experiments, we concentrated on those factors that were strongly induced and had already been linked to NK cell activation (IL-1β, IL-6, IL-8, IL-18, and MIP-1α) (72–78). In addition, IL-2, IL-12, IL-15, IL-21, and IFN-α/β, all known as NK cell stimulatory cytokines (7), were included in the analysis. IL-12p40 protein was detected in low amounts in the SNs of *Leishmania*-stimulated PBMCs but was absent when monocytes had been depleted (Figure 6F), indicating that it is released by *Leishmania*-triggered monocytes. The reason for the differential induction of IL-12p40 by the three *Leishmania* species (Figure 6F) tested is currently unknown, but is in line with previous observations that parasite species causing self-healing CL (*L. major*) elicit higher production of IL-12 as compared to parasite species associated with visceral disease (*L. donovani*) (79). Upregulation of CD69 on NK cells was clearly seen after stimulation of PBMCs with IL-2, IL-15, IL-18, or IFN-α/β, to a minor extent also with IL-1β and IL-6, but was not detectable following exposure of PBMCs to IL-8, IL-12, IL-21, or MIP-1α (Figure 7A). To determine whether one or several of the CD69-inducing cytokines represent the crucial NK cell-stimulatory component within the SNs of PBMC/*Leishmania* cocultures, SNs were preincubated with neutralizing Abs or respective isotype controls. We did not observe any alteration in NK cell CD69 expression upon addition of individual neutralizing Abs or combinations thereof, although the stimulatory effect of the respective recombinant cytokine was clearly abrogated by the Ab treatment (Figure 7B). We conclude that none of the CD69-inducing cytokines (IL-1β, IL-2, IL-12, IL-15, IL-18, and IFN-α/β) represents the soluble NK cell stimulating factor in supernatants of PBMC/*Leishmania* cocultures.

### IL-12 Is Required to Elicit NK Cell IFN-γ Release in Response to *Leishmania* in PBMC or NK/Monocyte Cultures

**Figure 8:**
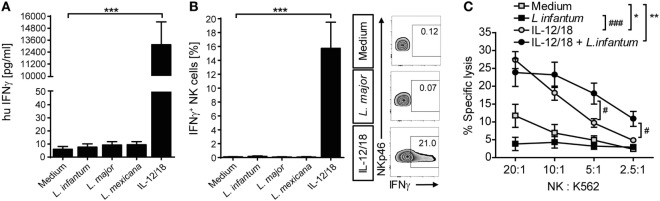
Coculture of peripheral blood mononuclear cells (PBMCs) with *Leishmania* neither induced natural killer (NK) cell interferon (IFN)-γ response nor NK cell cytotoxicity. Human PBMCs were cocultured with *Leishmania* spp. promastigotes (multiplicity of infection 10) and/or interleukin (IL)-12 and IL-18 (10 ng/ml each) for 20 h. IFN-γ production was determined by **(A,B)** ELISA of cell culture supernatants or **(B)** flow cytometry of intracellular IFN-γ in NKp46^+^CD3^−^ NK cells. **(C)** NK cell cytotoxicity was determined by measurement of specific lysis of ^51^Cr-labeled K562 tumor cells in a chromium-release assay. **(A)** Mean ± SEM of 76/71/55/46/74 donors for the five stimulations. **(B)** Mean ± SEM of 17/14/15/8/17 donors for the five stimulations; FACS plots show results of one representative donor. **(C)** Mean ± SEM of six (PBMC) or nine (PBMC + IL-12/18) donors. *^,#^*p* < 0.05; **^,##^*p* < 0.01; and ***^,###^*p* < 0.001 two-tailed Mann–Whitney *U* test.

Upregulation of CD69 is a first sign of NK cell activation but does not automatically entail the production of IFN-γ required for parasite control in mouse and human leishmaniasis (13). We therefore analyzed, whether IFN-γ was expressed by *Leishmania*-stimulated PBMCs. IFN-γ was neither detectable in culture SNs by ELISA (Figure 8A) nor in NK cells by intracellular cytokine staining (Figure 8B), whereas stimulation with IL-12/IL-18 elicited a clear IFN-γ response of NK cells (Figures 8A,B). Likewise, IL-12/IL-18, but not exposure to *Leishmania* enhanced the cytotoxic activity of NK cells (Figure 8C). Thus, stimulation of PBMCs by *Leishmania* was not sufficient to induce NK cell effector functions. Interestingly, stimulation of PBMCs with IL-12/18 and *Leishmania* promastigotes further increased NK cell cytotoxicity as compared with cells activated by IL-12/IL-18 alone (Figure 8C).

**Figure 9:**
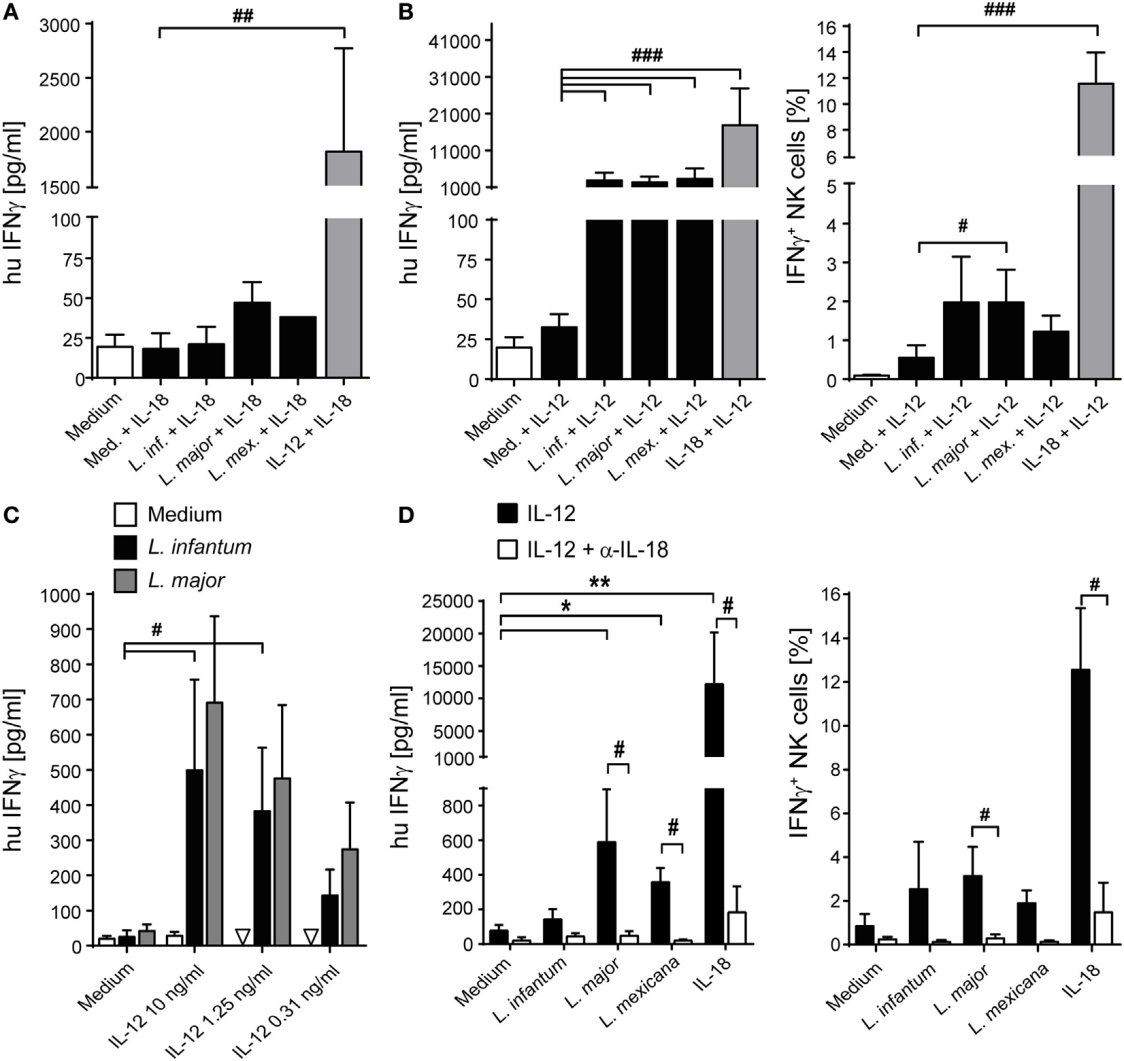
Effect of exogenous interleukin (IL)-12 and IL-18 on interferon (IFN)-γ production of natural killer (NK) cells in peripheral blood mononuclear cell (PBMC)-*Leishmania* cocultures. Human PBMCs were cocultured with *Leishmania* spp. promastigotes (multiplicity of infection 10) in presence or absence of exogenous IL-12 and/or IL-18 (10 ng/ml each, or as indicated) and neutralizing IL-18 antibody (1.5 µg/ml) for 20 h. Thereafter, IFN-γ production was measured either by ELISA of cell culture supernatants [**(A,B)** (left graph), **(C,D)** (left graph)] or by flow cytometry of intracellular IFN-γ in NKp46^+^CD3^−^ NK cells [**(B)** (right graph), **(D)** (right graph)]. Values below detection limit are marked by triangles. **(A)** Mean ± SEM of 6/6/6/3/1/6 donors for the six stimulations. **(B)** Mean ± SEM of 22/22/22/21/18/22 (ELISA, left panel) or 9 (ICS, right panel) donors. **(C)** Mean ± SEM of 6/6/3 donors for the three stimulations (medium, *Leishmania infantum*, and *Leishmania major*). **(D)** Mean ± SEM of six (ELISA, left panel) or five (ICS, right panel) donors. *^,#^*p* < 0.05, **^,##^*p* < 0.01, ^###^*p* < 0.001 two-tailed Mann–Whitney *U* test.

To determine whether exogenously added IL-12 and IL-18 differentially contributed to the NK cell effector response, PBMCs were cocultured with parasites in the presence of either IL-12 or IL-18. Whereas IL-18 was largely ineffective (Figure 9A), IL-12 and *Leishmania*, but not IL-12 alone, triggered the release of IFN-γ in PBMC cultures (Figure 9B). Titrating IL-12, a concentration of 300 pg/ml was sufficient to induce IFN-γ in the presence of *Leishmania* (Figure 9C). Blockade of IL-18 abolished the IL-12/*Leishmania*-induced IFN-γ response (Figure 9D).

**Figure 10:**
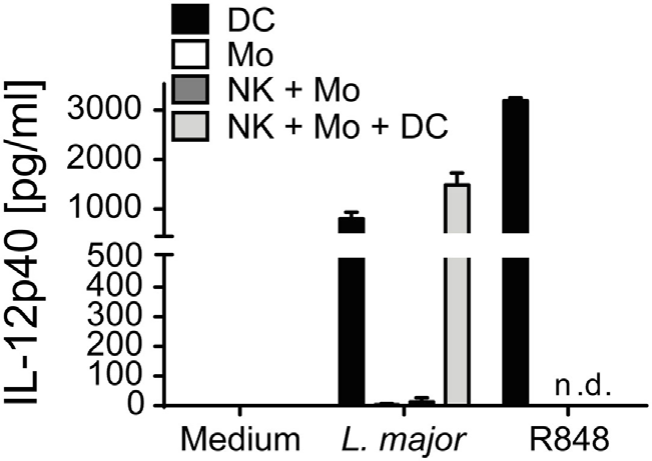
Blood CD1c^+^ dendritic cells (DCs) but not monocytes are a source of interleukin (IL)-12p40 in response to *Leishmania major*. Human blood CD1c^+^ DCs, CD14^+^ monocytes and NKp46^+^CD3^−^ natural killer (NK) cells were sorted and stimulated by *L. major* promastigotes (multiplicity of infection 10) for 20 h either alone or in combinations. R848 (5 µg/ml) was used as control. IL-12p40 content of the cell culture supernatants was measured by ELISA. Mean ± SEM of 4/4/3 donors for the three stimulations.

In the mouse system, DCs are IL-12 producers during the early phase of *Leishmania* infection (18, 80). To investigate whether primary human DCs are capable to respond to *Leishmania* parasites by secreting IL-12, CD1c^+^ DCs of human PBMCs were purified by cell sorting and cultured in the presence of *Leishmania* promastigotes. After 20 h, an average of 800 (±116) pg/ml IL-12p40 (mean ± SEM of four donors) was detected in the SNs of the DC cultures (Figure 10). By contrast, CD14^+^ monocytes sorted in parallel did not release measurable amounts of IL-12p40 in response to *Leishmania* (Figure 10). Interestingly, when CD1c^+^ DCs were incubated together with parasites and sorted monocytes and NK cells, the 20 h culture SNs contained an increased concentration of IL-12p40 (1,485 ± 213 pg/ml, mean ± SEM of four donors). Also, in the presence of all three host cells and *L. major* parasites low amounts of bioactive IL-12p70 (*ca*. 90 pg/ml) became detectable in two of four analyzed donors, whereas CD1c^+^ blood DCs or monocytes alone or cultures of monocytes and NK cells failed to generate IL-12p70 in response to *Leishmania* (data not shown).

Taken together, these data demonstrate that the absent NK cell effector response in PBMC/*Leishmania* cocultures is most likely due to an insufficient IL-12 production that presumably results from the low number of DCs in human PBMCs (68). Once IL-12 is added (or released by DCs after contact with NK cells and monocytes), endogenous IL-18 (produced and trans-presented by monocytes) acts synergistically with the IL-12 to elicit the expression of IFN-γ in NK cells.

### Expression of CD56 on Human NK Cells Is Downregulated after Contact with *Leishmania*

**Figure 11:**
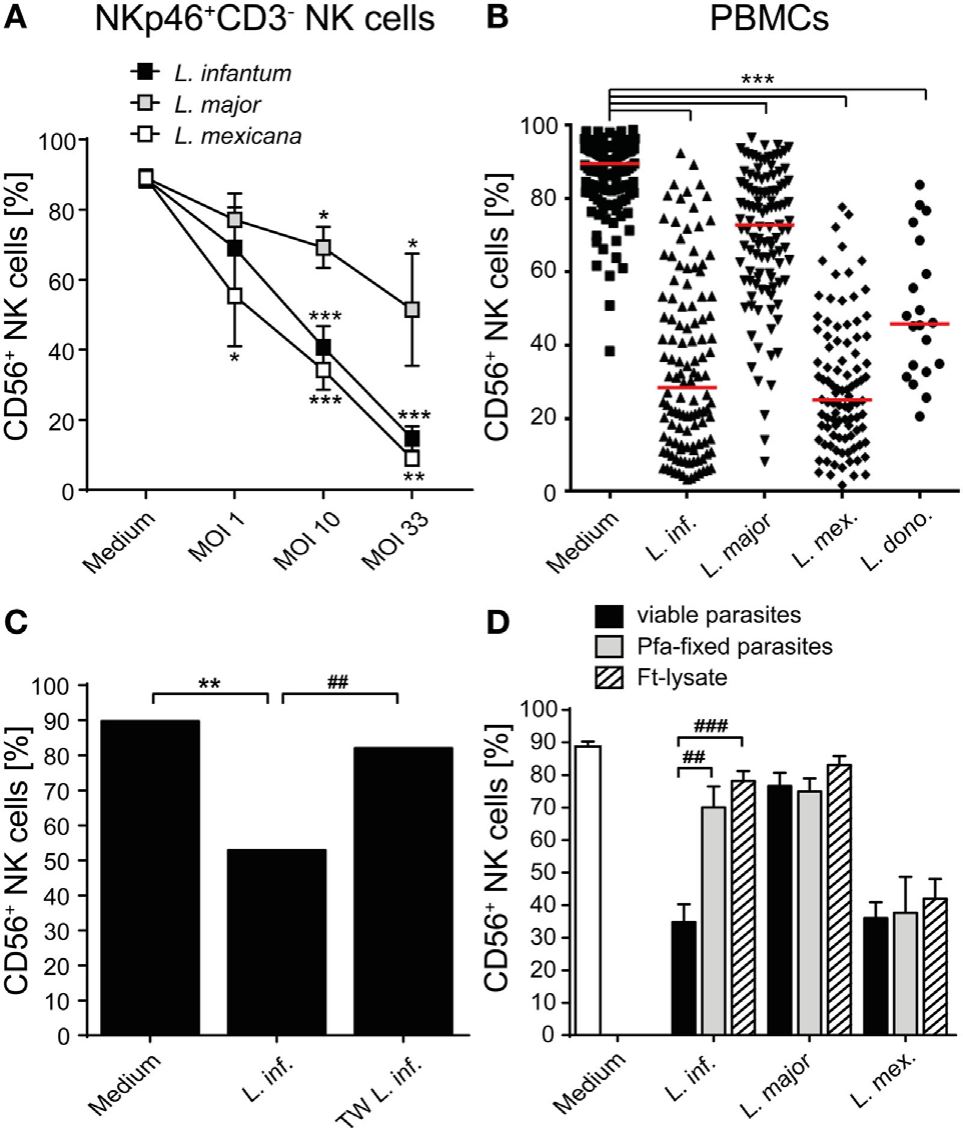
CD56 surface expression on natural killer (NK) cells is suppressed after contact with *Leishmania* promastigotes. **(A)** Purified NKp46^+^/CD3^−^ NK cells or **(B–D)** human peripheral blood mononuclear cells (PBMCs) were cocultured with viable **(A–D)**, paraformaldehyde (Pfa)-fixed **(D)**, or freeze–thaw-lysed **(D)** *Leishmania* spp. promastigotes [multiplicity of infection (MOI) 10 unless otherwise indicated] for 20 h. CD56 surface expression on NKp46^+^/CD3^−^ NK cells was measured by flow cytometry. **(A)** Mean ± SEM of 17/16/12/12 donors for the four stimulations. **(B)** Mean ± SEM of 132/121/114/97/20 donors for the five stimulations. **(C)** Mean ± SEM of 6/6/6 donors for the three stimulations [transwell (TW)]. **(D)** Mean ± SEM of 22 (medium), 22/17/18 (untreated and viable parasites), 8/9/6 (Pfa-fixed parasites), or 17/14/14 [freeze–thaw lysate (ft-lysate)] donors. *^,#^*p* < 0.05; **^,##^*p* < 0.01; an ***^,###^*p* < 0.001 two-tailed Mann–Whitney *U* test.

**Figure 12:**
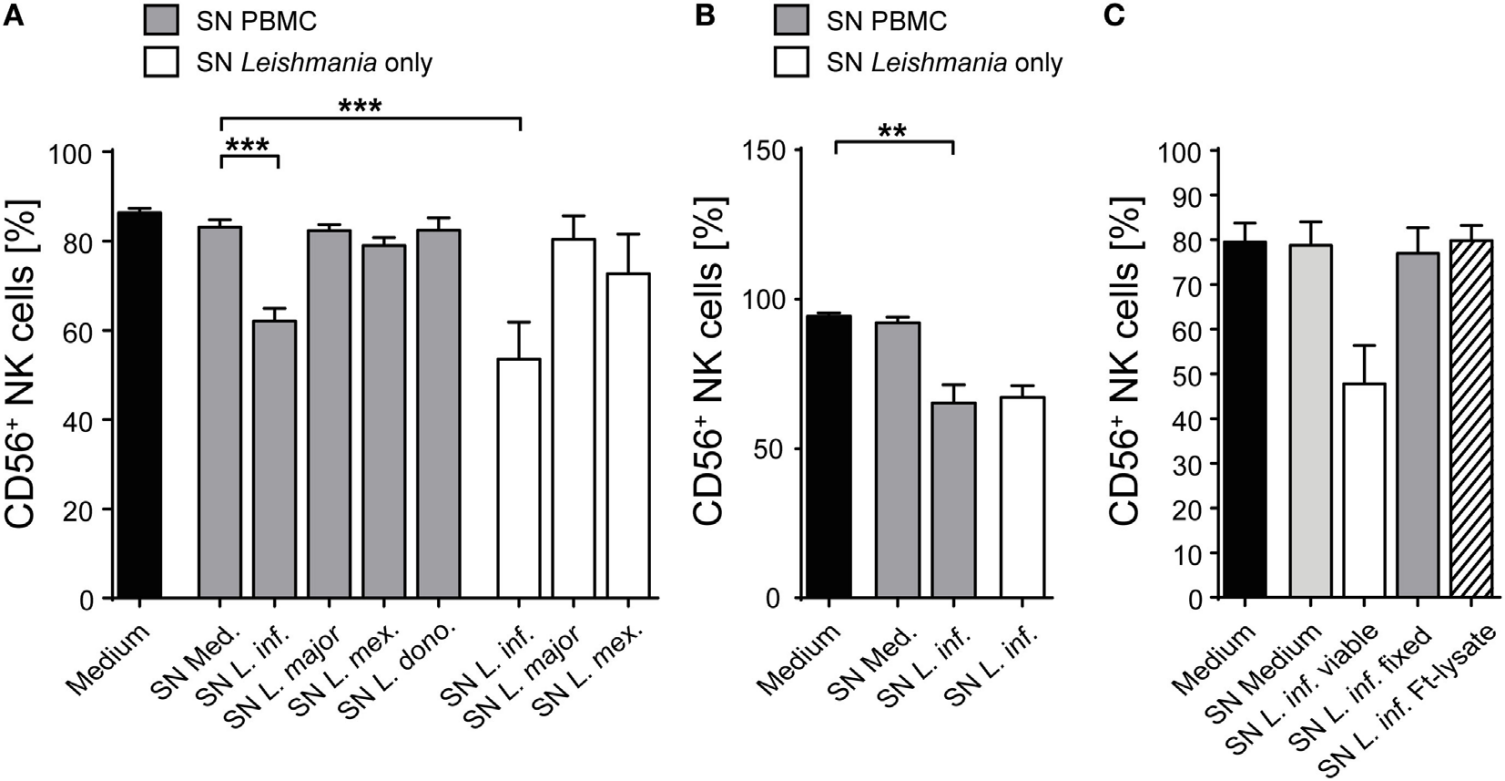
Supernatants (SNs) from peripheral blood mononuclear cell (PBMC)/*Leishmania infantum* cocultures downmodulate CD56 expression on natural killer (NK) cells. SNs from PBMC/*Leishmania* cocultures (multiplicity of infection 10) or from pure *Leishmania* cultures were used at a concentration of 60% (vol/vol) to stimulate **(A,C)** PBMCs or **(B)** purified NKp46^+^/CD3^−^ NK cells for 20 h. CD56 surface expression on NKp46^+^/CD3^−^ NK cells was determined by flow cytometry. **(A)** Mean ± SEM of 61 (medium), 59/59/53/50/15 (SN PBMC/*Leishmania*-coculture), or 8/8/6 (SN *Leishmania* only) donors. **(B)** Mean ± SEM of eight (medium), eight (SN PBMC/*Leishmania*-coculture), or two (SN *Leishmania* only) donors. **(C)** Mean ± SEM of 8/8/8/7/5 donors for the five stimulations. **p < 0.01; and ***p < 0.001 two-tailed Mann–Whitney *U* test.

When analyzing the activation of NK cells within human PBMCs or of sorted NKp46^+^CD3^−^ human NK cells after coculture with *Leishmania* promastigotes, we noticed that the surface expression of the NK cell marker CD56 (NCAM1), whose functional role is still unknown, was reduced. The downregulation of CD56 was dependent on the parasite/host cell ratio and the parasite species, with *L. infantum* and *L. mexicana* causing more pronounced effects than *L. donovani* or *L. major* (Figures 11A,B). The decrease of CD56 was observed in both the CD56^bright^ and CD56^dim^ NK cell population (data not shown; see also below in Figure 14, panel “medium” vs. “*L. infantum*”). Separation of NK cells and *Leishmania* using a TW culture system abolished the effect (Figure 11C), indicating that direct contact between NK cells and parasites was required. To exclude that *Leishmania* (products) occupy certain epitopes on NK cells and thereby prevent the detection of CD56, we tested different monoclonal Abs against human CD56 (clones CMSSB, HCD56, and MEM-188), all of which yielded similar results (data not shown). For *L. infantum*, the decrease in CD56 expression was not observed when fixed or lysed instead of viable parasites were used, whereas the downregulatory effect of *L. major* or *L. mexicana* promastigotes on CD56 was maintained even after lysis or fixation of the parasites (Figure 11D). A limited, but still significant suppression of CD56 was also seen with SNs from pure parasite cultures or from PBMC/*Leishmania* cocultures, but only in the case of viable *L. infantum* and not with any of the other *Leishmania* species (Figures 12A–C).

**Figure 13:**
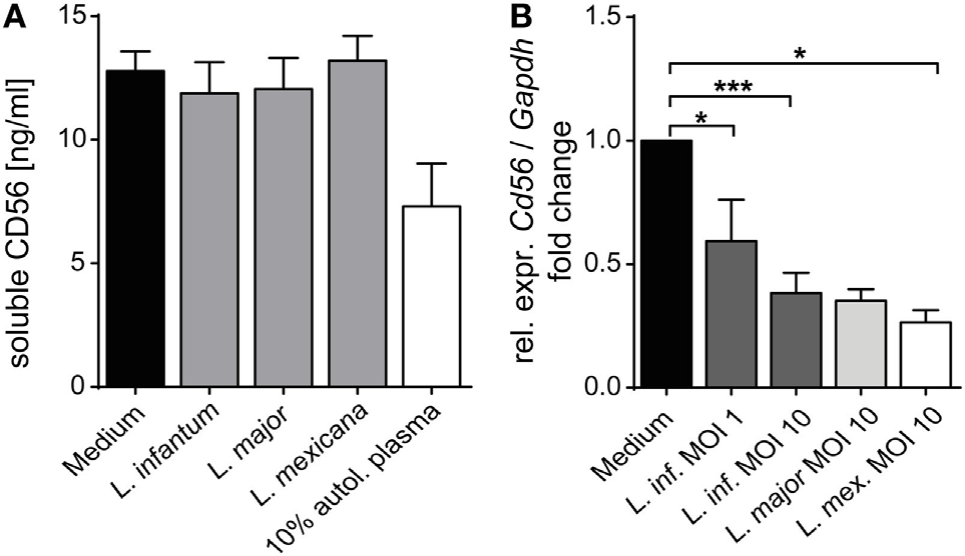
Mechanism of *Leishmania*-induced reduction of surface CD56 on natural killer (NK) cells. **(A)** Purified human NKp46^+^CD3^−^ NK cells were cocultured with *Leishmania* promastigotes [multiplicity of infection (MOI) 10] for 20 h. The concentration of soluble CD56 in the cell culture supernatants was determined by ELISA. Mean ± SEM of 9/7/6/7/3 donors for the five stimulations. **(B)** Purified human NKp46^+^CD3^−^ NK cells were cocultured with *Leishmania* promastigotes at different parasite/host cell ratios for 20 h. mRNA expression of CD56 was quantified by TaqMan RT-PCR. The expression was normalized against the endogenous control (huGAPDH), and the fold change was calibrated to the respective medium value. Mean ± SEM of 8/3/8/2/5 donors for the five stimulations. *p < 0.05, **p < 0.01, and ***p < 0.001 two-tailed Mann–Whitney *U* test.

As CD56 also exists in a soluble form which is directly secreted or released from the cell surface (81, 82), we considered the possibility that *Leishmania* parasites induce CD56 shedding by NK cells. However, the concentration of soluble CD56 detected in SNs of purified NK cell/*Leishmania* spp. cocultures was comparable to the amount of sCD56 found in SNs of NK cell cultures without parasites (Figure 13A). Instead, exposure of sorted Nkp46^+^CD3^−^ NK cells to *Leishmania* caused a significant reduction of CD56 mRNA in a dose-dependent manner (Figure 13B). Thus, *Leishmania*-induced transcriptional suppression of CD56 mRNA rather than shedding of CD56 surface protein accounts for the decrease in CD56^+^ NK cells after contact with *Leishmania*.

**Figure 14:**
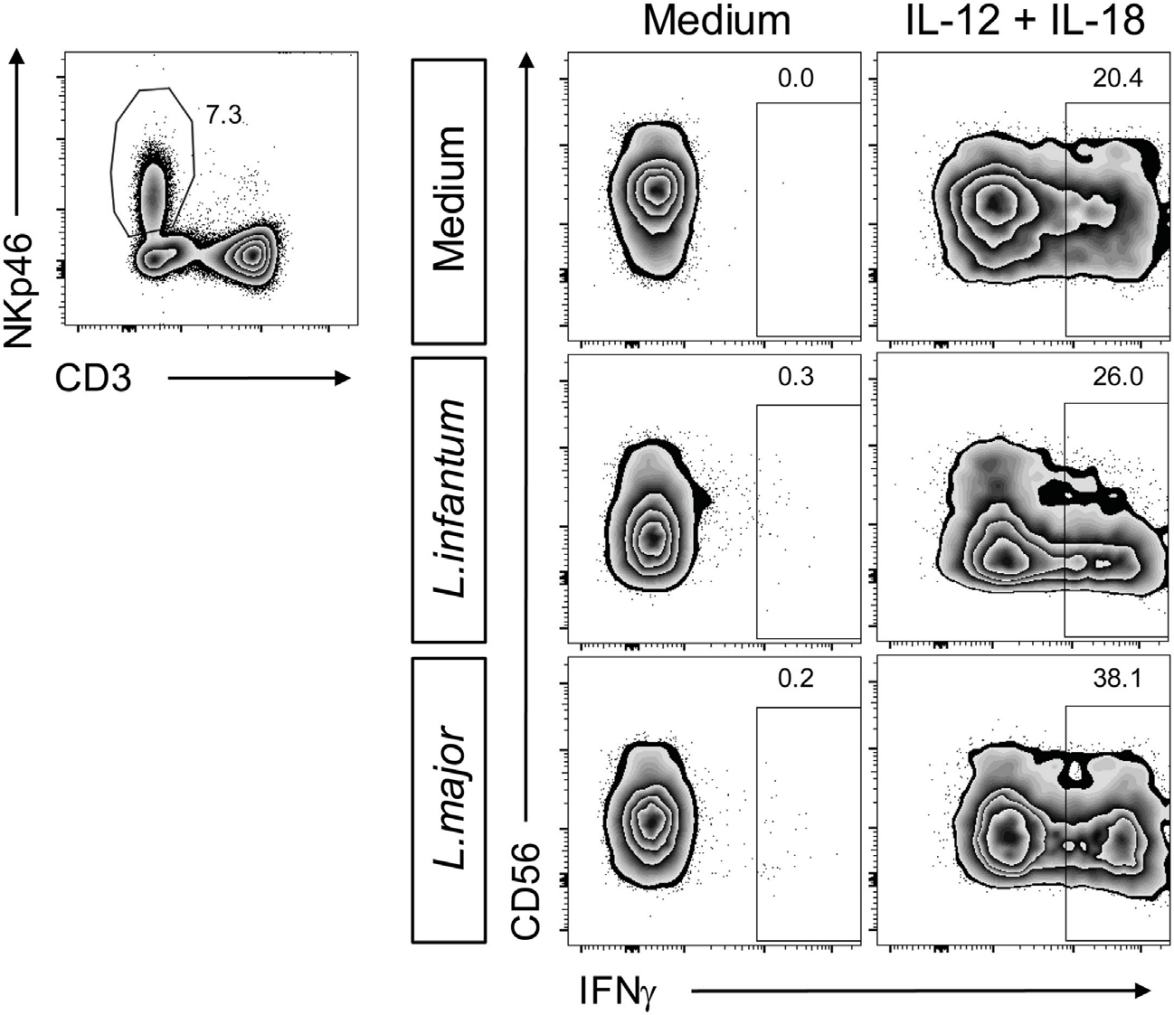
Suppression of natural killer (NK) cell CD56 expression by *Leishmania* does not affect cytokine-induced interferon (IFN)-γ production. Human peripheral blood mononuclear cells were stimulated by *Leishmania* promastigotes (multiplicity of infection 10) in the absence or presence of interleukin (IL)-12 and IL-18 (10 ng/ml each) for 20 h. Intracellular IFN-γ in NKp46^+^CD3^−^ NK cells was measured by flow cytometry. Results of one of two donors analyzed are shown.

Finally, we addressed the question, whether the *Leishmania*-induced down-modulation of CD56 influences NK cell cytokine responsiveness. Therefore, PBMCs were simultaneously exposed to *Leishmania* and IL-12/18. As under these conditions the IFN-γ production by NK cells was higher as with cytokine stimulation alone and CD56^dim/negative^ NK cells turned out to be strong IFN-γ producers (Figure 14), we conclude that a lack of CD56 does not hamper NK cell activation by IL-12/IL-18.

## Discussion

Human blood NK cells will be in contact with *Leishmania* promastigotes during the first hours of natural infection, as the sand flies vectors regurgitate the parasites into a blood pool generated by laceration of skin capillaries. Previous analyses of the effector responses of human blood NK cells to *Leishmania* parasites yielded controversial results and did not identify the host-derived signals required for NK cell activation [reviewed in Ref. (13); see discussion below]. In this study, we investigated the effect of different parasite species and strains and aimed to define the cellular and humoral prerequisites for *Leishmania*-induced NK cell activation. We used blood NK cells of volunteers from a non-endemic area that were incubated with *Leishmania* promastigotes for 20 h in the presence of 10% autologous plasma, thus mimicking the early phase of infection and the microenvironment of a naïve host during primary infection.

### CD69 and Activation of Human Blood NK Cells by *Leishmania*: Monocyte Contact Dependent vs. Soluble Signals

Our experiments revealed that efficient upregulation of the early activation marker CD69 on *Leishmania*-stimulated NK cells required (a) cell–cell contact between NK cells and monocytes and (b) a soluble, heat-labile factor released by infected monocytes. This two-signal model is based on the observations that on the one hand upregulation of CD69 was prevented following physical separation of NK cells and monocytes, whereas on the other hand native, but not heat-treated SNs of cocultures of viable *Leishmania* promastigotes with monocytes were able to induce CD69 expression on NK cells. The findings were true for all *Leishmania* species tested [*L. infantum* (dermotropic and viscerotropic strains), *L. major, L. mexicana*, and *L. donovani*], although the extent of CD69 upregulation varied between the numerous donors analyzed and differed significantly between *Leishmania* species.

Several results obtained in this study strongly support the idea that IL-18 trans-presented by infected monocytes to NK cells constitutes the contact-dependent signal: (i) IL-18 was detectable on the surface of non-permeabilized monocytes after exposure to *Leishmania*; (ii) culture SNs of infected monocytes contained little or no IL-18; and (iii) neutralizing Abs to IL-18 largely prevented CD69 induction on NK cells when added directly to PBMC/*Leishmania* cultures, but did not abolish the CD69-inducing activity of SNs from previous PBMC/*Leishmania* cultures. The available data, however, do not formally exclude the possibility that pro-IL-18, which lacks a secretory leader sequence, is nevertheless locally released into synapses between NK cells and monocytes *via* directed exocytosis of secretory lysosomes as described for NK/DC interactions (83) and that anti-IL-18 is able to access and neutralize IL-18 in such a scenario.

With respect to the soluble factor, which is heat-labile and awaits further characterization, the NK cell-activating cytokines IFN-α/β, IL-1β, IL-2, IL-6, IL-8, IL-12, IL-15, IL-18, IL-21, and MIP-1α were excluded to account for the CD69-upregulating effect of the culture SNs. At this stage, we cannot rule out that the soluble factor is a *Leishmania*-derived protein that is only released by infected monocytes [e.g., *via* exosomes (84)], but not by the parasite itself, as SNs of pure *Leishmania* cultures had no effect on CD69 expression of NK cells. Considering that culture SNs of *Leishmania*-infected monocytes remained active after passage through a 0.22 µm sterile filter, it was unexpected that NK cell activation was completely prevented when monocytes and *Leishmania* were separated from NK cells by a membrane with 0.4 µm pore size using a TW system. A plausible explanation is that in case of the culture SNs the starting concentration of the unknown factor is much higher (as it accumulated over 20 h), whereas in the TW setting the factor is newly produced and only slowly builds up.

### CD69 and Activation of Human Blood NK Cells by *Leishmania*: Direct vs. Indirect Stimulation

The observation that myeloid cells were necessary to activate human NK cells by *Leishmania* is in line with our observations in murine leishmaniasis (18, 49, 80). There are also a few previous studies, in which human blood NK cells alone failed to respond to *Leishmania* antigen (57) and required the presence of adherent PBMCs (58, 59); however, in these reports only proliferation and cytokine production of NK cells, but not their expression of CD69 were analyzed. On the other hand, our current results clearly contrast with earlier findings that viable or dead promastigotes of *Leishmania aethiopica, L. mexicana*, and *L. donovani* directly triggered IFN-γ release by sorted NK cells (60, 61). In one of these studies purified *Leishmania* LPG was claimed to directly bind to TLR2 on NK cells (61), whereas Nylen et al. (60) found that LPG-deficient *L. mexicana* mutants were as potent as wild-type parasites in activating NK cells. Notably, the same group later failed to recapitulate accessory cell-independent NK cell activation by *Leishmania* using two different strains of *L. major* (85). Possible explanations for the lack of direct NK cell stimulation by *Leishmania* in our hands are (a) a higher degree of NK cell purity [>96 vs. 90% (60)], (b) a shorter stimulation period [20 vs. 48 h (60)], and/or (c) the use of blood cells from donors of a non-endemic vs. endemic area (61).

The variable degree of monocyte-dependent NK cell activation we have seen with the four tested *Leishmania* species might reflect species-specific differences in the monocyte/parasite interaction. For example, *L. infantum* promastigotes were less efficiently phagocytosed by human monocytes than *L. major* promastigotes (86), whereas twice as many *L. major* promastigotes were needed to achieve the same infection rate in monocytes as with *L. donovani* (87). Thus, the differential uptake of *Leishmania* parasites by monocytes exactly correlates with our results on the upregulation of CD69 on NK cells (*L. donovani* > *L. major* > *L. infantum*). The possibility that differences in CD69 induction are indeed determined by the infection rate of monocytes is further supported by three observations. First, higher parasite/host cell ratios were associated with an increased percentage of CD69^+^ NK cells in the culture. Second, fixed parasites, which are morphologically intact and therefore likely engage phagocytosis-accelerating receptors (36), were more potent in upregulating CD69 than parasite lysates. Third, non-classical monocytes, which tentatively showed the weakest effect on CD69 induction, were reported to exhibit low phagocytic activity (88).

### NK Cell Effector Response upon Stimulation by *Leishmania*

Despite induction of CD69 neither NK cells within *Leishmania*-stimulated PBMCs nor purified NK cells cocultured with infected monocytes produced IFN-γ or showed an upregulation of cytotoxic activity. However, the parasites acted as costimulus by augmenting IL-12/IL-18-induced NK cytotoxicity in PBMC cultures. Furthermore, NK cells within PBMC/*Leishmania* cultures were capable to secrete IFN-γ following addition of exogenous IL-12 (≥300 pg/ml). This result is in accordance with previous observations reporting a lack of IFN-γ production by NK cells in pure PBMC/promastigote cocultures (62, 63) or in human NK/DC cocultures after neutralization of IL-12 (89). Furthermore, it is known that activation of NK cell effector responses frequently requires cooperation between cytokines (e.g., IL-12 and IL-2 or IL-15; IL-2 and IL-15; IL-12 and IL-18) (25, 49, 90–92). Especially IL-18 was shown to prime NK cells to become responsive to IL-12 (16, 83, 93). Our results on IL-12- and *Leishmania*-mediated induction of IFN-γ secretion by human NK cells represents a further example of the cooperative interaction between IL-12 and endogenously generated IL-18 and confirm our findings in the mouse (18, 49).

Interleukin-12 production in pure PBMC/*Leishmania* promastigote cocultures was ineffective. Infected monocytes produced only low amounts of IL-12 during the 20 h culture period, and DCs, which can release IL-12 in response to *Leishmania* (79, 94), are rare in PBMCs (68). Primary CD1c^+^ blood DCs stimulated by *L. major* promastigotes secreted IL-12p40 without any further maturation signal. In contrast to previous work (94), the sorted CD1c^+^ DCs reacted to promastigotes and not only to amastigotes. Coculture of CD1c^+^ DC with monocytes, NK cells and *L. major* even led to the detection of IL-12p70. As CD1c^+^ DCs are also present in the skin (95), the primary site of *Leishmania* infection, they likely become activated during the early immune reaction and might contribute to NK cell activation *in situ* by release of IL-12. Also, in secondary lymphoid organs DCs were reported to colocalize with NK cells (89). Thus, close interaction between NK cells, DCs, and infiltrating monocytes during *Leishmania* infection appears plausible also in other organs such as spleen and liver, which are main targets of the parasite in VL.

### Suppression of CD56 by *Leishmania*

Direct contact of *Leishmania* promastigotes with NK cells caused reduction of CD56 mRNA and protein. While the decrease in CD56 mRNA was comparable for *L. major, L. infantum*, and *L. mexicana*, surface CD56 was less strongly downregulated by *L. major* as compared with *L. infantum* and *L. mexicana*. Differential secretion or shedding of CD56 was excluded. However, as NK cells are able to export CD56 in exosomes (96), the divergent regulation of CD56 protein might result from *Leishmania* species-specific induction of CD56^+^ exosomes and their release by NK cells.

Reduction of CD56 on human NK cells in response to *L. major* was previously observed by Lieke et al. (85). Compared with our results the effect was much stronger, which might reflect the use of different *L. major* strains. Lieke et al. postulated that the *Leishmania* surface protease gp63 was critical for the suppression of CD56 protein on NK cells (85). However, this hypothesis remains questionable, as in NK/*Leishmania* cocultures gp63-deficient parasites lacked the downregulation of CD56 only after 24 h, but not after 120 h of coculture when the effect of wild-type and knockout parasites was comparable. As gp63 is produced by all *Leishmania* species, it is unlikely to account for the differential regulation of CD56 by the *Leishmania* species tested in our study.

Contact-dependent reduction in CD56 surface expression on human NK cells in response to pathogens is not restricted to *Leishmania* parasites, but was also observed in cocultures of NK cells with *Aspergillus* spp. (97), suggesting that it might be a more general evasion mechanism. Our knowledge of the function of CD56 expressed by human NK cells is still limited. Several publications showed upregulation of CD56 upon activation of human NK cells; conversely, downregulation of CD56 was reported when NK cells had been exposed to an immunosuppressive milieu (35). It is not yet clear whether CD56 only represents a marker of an activated cell state or is directly involved in immune effector functions. Recently, CD56 was found to play a role in NK cell maturation, as it facilitated migration of NK cells on stromal cells (98). Also, homophilic interactions between CD56 molecules on CD56^+^ NK cells and CD56^+^ target cells triggered NK cell cytotoxicity (99). Viral infections and autoimmune diseases were reported to give rise to dysfunctional CD56-negative NK cells, which showed reduced activity even after stimulation with cytokines (35). In our study, however, NK cell IFN-γ release in response to activating cytokines and *Leishmania* parasites was not impaired, despite the downregulation of CD56.

## Ethics Statement

The work with human cells from normal donors had been approved by the Ethics Committee of the Friedrich-Alexander-University (FAU) Erlangen-Nürnberg (approval no. 112_12 B and 185_12 B). Informed written consents were obtained in accordance with the Declaration of Helsinki.

## Author Contributions

Conceived and designed the experiments: US, HM, DD, and CB. Performed the experiments: HM, HS, and LH. Analyzed the data: HM, HS, LH, DD, CB, and US. Wrote the paper: CB, HM, and US.

## Conflict of Interest Statement

The authors declare that the research was conducted in the absence of any commercial or financial relationships that could be construed as a potential conflict of interest.

## Abbreviations

DC, dendritic cell; NK, natural killer; IFN, interferon; ILC, innate lymphoid cell; ft-lysate, freeze–thaw lysate; PBMC, peripheral blood mononuclear cell; Pfa, paraformaldehyde; Th, T helper lymphocytes; TW, transwell.

## References

[1] Eberl G Colonna M Di Santo JP McKenzie AN. Innate lymphoid cells. Innate lymphoid cells: a new paradigm in immunology. Science (2015) 348(6237):aaa6566. 10.1126/science.aaa6566 25999512 PMC5658207

[2] Borrego F Pena J Solana R. Regulation of CD69 expression on human natural killer cells: differential involvement of protein kinase C and protein tyrosine kinases. Eur J Immunol (1993) 23(5):1039–43. 10.1002/eji.1830230509 8477800

[3] Blaser C Kaufmann M Pircher H. Virus-activated CD8 T cells and lymphokine-activated NK cells express the mast cell function-associated antigen, an inhibitory C-type lectin. J Immunol (1998) 161(12):6451–4. 9862665

[4] Lanier LL Buck DW Rhodes L Ding A Evans E Barney C Interleukin 2 activation of natural killer cells rapidly induces the expression and phosphorylation of the Leu-23 activation antigen. J Exp Med (1988) 167(5):1572–85. 10.1084/jem.167.5.1572 3259252 PMC2188935

[5] Gerosa F Tommasi M Benati C Gandini G Libonati M Tridente G Differential effects of tyrosine kinase inhibition in CD69 antigen expression and lytic activity induced by rIL-2, rIL-12, and rIFN-alpha in human NK cells. Cell Immunol (1993) 150(2):382–90. 10.1006/cimm.1993.1206 8103709

[6] Pisegna S Zingoni A Pirozzi G Cinque B Cifone MG Morrone S Src-dependent Syk activation controls CD69-mediated signaling and function on human NK cells. J Immunol (2002) 169(1):68–74. 10.4049/jimmunol.169.1.68 12077230

[7] Vivier E Raulet DH Moretta A Caligiuri MA Zitvogel L Lanier LL Innate or adaptive immunity? The example of natural killer cells. Science (2011) 331(6013):44–9. 10.1126/science.1198687 21212348 PMC3089969

[8] Lopez-Soto A Gonzalez S Smyth MJ Galluzzi L. Control of metastasis by NK cells. Cancer Cell (2017) 32(2):135–54. 10.1016/j.ccell.2017.06.009 28810142

[9] Vivier E Tomasello E Baratin M Walzer T Ugolini S. Functions of natural killer cells. Nat Immunol (2008) 9(5):503–10. 10.1038/ni1582 18425107

[10] Li SS Kyei SK Timm-McCann M Ogbomo H Jones GJ Shi M The NK receptor NKp30 mediates direct fungal recognition and killing and is diminished in NK cells from HIV-infected patients. Cell Host Microbe (2013) 14(4):387–97. 10.1016/j.chom.2013.09.007 24139398

[11] Lieke T Graefe SE Klauenberg U Fleischer B Jacobs T. NK cells contribute to the control of *Trypanosoma cruzi* infection by killing free parasites by perforin-independent mechanisms. Infect Immun (2004) 72(12):6817–25. 10.1128/IAI.72.12.6817-6825.2004 15557602 PMC529106

[12] Small CL McCormick S Gill N Kugathasan K Santosuosso M Donaldson N NK cells play a critical protective role in host defense against acute extracellular *Staphylococcus aureus* bacterial infection in the lung. J Immunol (2008) 180(8):5558–68. 10.4049/jimmunol.180.8.5558 18390740

[13] Bogdan C. Natural killer cells in experimental and human leishmaniasis. Front Cell Infect Microbiol (2012) 2:69. 10.3389/fcimb.2012.00069 22919660 PMC3417408

[14] Bukowski JF Woda BA Habu S Okumura K Welsh RM. Natural killer cell depletion enhances virus synthesis and virus-induced hepatitis in vivo. J Immunol (1983) 131(3):1531–8. 6309965

[15] Baratin M Roetynck S Lepolard C Falk C Sawadogo S Uematsu S Natural killer cell and macrophage cooperation in MyD88-dependent innate responses to *Plasmodium falciparum*. Proc Natl Acad Sci U S A (2005) 102(41):14747–52. 10.1073/pnas.0507355102 16203971 PMC1253601

[16] Chaix J Tessmer MS Hoebe K Fuseri N Ryffel B Dalod M Cutting edge: priming of NK cells by IL-18. J Immunol (2008) 181(3):1627–31. 10.4049/jimmunol.181.3.1627 18641298 PMC5154249

[17] Lucas M Schachterle W Oberle K Aichele P Diefenbach A. Dendritic cells prime natural killer cells by trans-presenting interleukin 15. Immunity (2007) 26(4):503–17. 10.1016/j.immuni.2007.03.006 17398124 PMC2084390

[18] Schleicher U Liese J Knippertz I Kurzmann C Hesse A Heit A NK cell activation in visceral leishmaniasis requires TLR9, myeloid DCs, and IL-12, but is independent of plasmacytoid DCs. J Exp Med (2007) 204(4):893–906. 10.1084/jem.20061293 17389237 PMC2118560

[19] Guia S Cognet C de Beaucoudrey L Tessmer MS Jouanguy E Berger C A role for interleukin-12/23 in the maturation of human natural killer and CD56+ T cells in vivo. Blood (2008) 111(10):5008–16. 10.1182/blood-2007-11-122259 18319400

[20] Trinchieri G Santoli D. Anti-viral activity induced by culturing lymphocytes with tumor-derived or virus-transformed cells. Enhancement of human natural killer cell activity by interferon and antagonistic inhibition of susceptibility of target cells to lysis. J Exp Med (1978) 147(5):1314–33. 10.1084/jem.147.5.1314 650156 PMC2184280

[21] Mori S Jewett A Cavalcanti M Murakami-Mori K Nakamura S Bonavida B. Differential regulation of human NK cell-associated gene expression following activation by IL-2, IFN-alpha and PMA/ionomycin. Int J Oncol (1998) 12(5):1165–70. 9538144 10.3892/ijo.12.5.1165

[22] Cooper MA Fehniger TA Ponnappan A Mehta V Wewers MD Caligiuri MA. Interleukin-1beta costimulates interferon-gamma production by human natural killer cells. Eur J Immunol (2001) 31(3):792–801. 10.1002/1521-4141(200103)31:3<792::AID-IMMU792>3.0.CO;2-U 11241284

[23] Trinchieri G Matsumoto-Kobayashi M Clark SC Seehra J London L Perussia B. Response of resting human peripheral blood natural killer cells to interleukin 2. J Exp Med (1984) 160(4):1147–69. 10.1084/jem.160.4.1147 6434688 PMC2187474

[24] Chehimi J Valiante NM D’Andrea A Rengaraju M Rosado Z Kobayashi M Enhancing effect of natural killer cell stimulatory factor (NKSF/interleukin-12) on cell-mediated cytotoxicity against tumor-derived and virus-infected cells. Eur J Immunol (1993) 23(8):1826–30. 10.1002/eji.1830230814 8102101

[25] Fehniger TA Shah MH Turner MJ VanDeusen JB Whitman SP Cooper MA Differential cytokine and chemokine gene expression by human NK cells following activation with IL-18 or IL-15 in combination with IL-12: implications for the innate immune response. J Immunol (1999) 162(8):4511–20. 10201989

[26] Skak K Frederiksen KS Lundsgaard D. Interleukin-21 activates human natural killer cells and modulates their surface receptor expression. Immunology (2008) 123(4):575–83. 10.1111/j.1365-2567.2007.02730.x 18005035 PMC2433320

[27] Ziblat A Domaica CI Spallanzani RG Iraolagoitia XL Rossi LE Avila DE IL-27 stimulates human NK-cell effector functions and primes NK cells for IL-18 responsiveness. Eur J Immunol (2015) 45(1):192–202. 10.1002/eji.201444699 25308526

[28] Long EO Kim HS Liu D Peterson ME Rajagopalan S. Controlling natural killer cell responses: integration of signals for activation and inhibition. Annu Rev Immunol (2013) 31:227–58. 10.1146/annurev-immunol-020711-075005 23516982 PMC3868343

[29] Walzer T Blery M Chaix J Fuseri N Chasson L Robbins SH Identification, activation, and selective in vivo ablation of mouse NK cells via NKp46. Proc Natl Acad Sci U S A (2007) 104(9):3384–9. 10.1073/pnas.0609692104 17360655 PMC1805551

[30] Cooper MA Fehniger TA Fuchs A Colonna M Caligiuri MA. NK cell and DC interactions. Trends Immunol (2004) 25(1):47–52. 10.1016/j.it.2003.10.012 14698284

[31] Fehniger TA Cooper MA Nuovo GJ Cella M Facchetti F Colonna M CD56bright natural killer cells are present in human lymph nodes and are activated by T cell-derived IL-2: a potential new link between adaptive and innate immunity. Blood (2003) 101(8):3052–7. 10.1182/blood-2002-09-2876 12480696

[32] Fauriat C Long EO Ljunggren HG Bryceson YT. Regulation of human NK-cell cytokine and chemokine production by target cell recognition. Blood (2010) 115(11):2167–76. 10.1182/blood-2009-08-238469 19965656 PMC2844017

[33] De Maria A Bozzano F Cantoni C Moretta L. Revisiting human natural killer cell subset function revealed cytolytic CD56(dim)CD16+ NK cells as rapid producers of abundant IFN-gamma on activation. Proc Natl Acad Sci U S A (2011) 108(2):728–32. 10.1073/pnas.1012356108 21187373 PMC3021076

[34] Ellis TM Fisher RI Functional heterogeneity of Leu 19“bright”+ and Leu 19“dim”+ lymphokine-activated killer cells. J Immunol (1989) 142(8):2949–54. 2467946

[35] Van Acker HH Capsomidis A Smits EL Van Tendeloo VF. CD56 in the immune system: more than a marker for cytotoxicity? Front Immunol (2017) 8:892. 10.3389/fimmu.2017.00892 28791027 PMC5522883

[36] Kaye P Scott P. Leishmaniasis: complexity at the host-pathogen interface. Nat Rev Microbiol (2011) 9(8):604–15. 10.1038/nrmicro2608 21747391

[37] Bogdan C. Leishmaniasis in rheumatology, haematology and oncology: epidemiological, immunological and clinical aspects and caveats. Ann Rheum Dis (2012) 71(Suppl 2):i60–6. 10.1136/annrheumdis-2011-200596 22460140

[38] Bogdan C. Mechanisms and consequences of persistence of intracellular pathogens: leishmaniasis as an example. Cell Microbiol (2008) 10(6):1221–34. 10.1111/j.1462-5822.2008.01146.x 18363880

[39] Wilson ME Jeronimo SM Pearson RD. Immunopathogenesis of infection with the visceralizing *Leishmania* species. Microb Pathog (2005) 38(4):147–60. 10.1016/j.micpath.2004.11.002 15797810

[40] Sacks D Noben-Trauth N. The immunology of susceptibility and resistance to *Leishmania major* in mice. Nat Rev Immunol (2002) 2(11):845–58. 10.1038/nri933 12415308

[41] Melby PC Andrade-Narvaez FJ Darnell BJ Valencia-Pacheco G Tryon VV Palomo-Cetina A. Increased expression of proinflammatory cytokines in chronic lesions of human cutaneous leishmaniasis. Infect Immun (1994) 62(3):837–42. 8112853 10.1128/iai.62.3.837-842.1994 PMC186190

[42] Valencia-Pacheco G Loria-Cervera EN Sosa-Bibiano EI Canche-Pool EB Vargas-Gonzalez A Melby PC In situ cytokines (IL-4, IL-10, IL-12, IFN-gamma) and chemokines (MCP-1, MIP-1alpha) gene expression in human *Leishmania* (*Leishmania*) *mexicana* infection. Cytokine (2014) 69(1):56–61. 10.1016/j.cyto.2014.05.016 25022962

[43] Ghalib HW Piuvezam MR Skeiky YA Siddig M Hashim FA el-Hassan AM Interleukin 10 production correlates with pathology in human *Leishmania donovani* infections. J Clin Invest (1993) 92(1):324–9. 10.1172/JCI116570 8326000 PMC293600

[44] Singh OP Stober CB Singh AK Blackwell JM Sundar S. Cytokine responses to novel antigens in an Indian population living in an area endemic for visceral leishmaniasis. PLoS Negl Trop Dis (2012) 6(10):e1874. 10.1371/journal.pntd.0001874 23150744 PMC3493615

[45] Peruhype-Magalhaes V Martins-Filho OA Prata A Silva Lde A Rabello A Teixeira-Carvalho A Immune response in human visceral leishmaniasis: analysis of the correlation between innate immunity cytokine profile and disease outcome. Scand J Immunol (2005) 62(5):487–95. 10.1111/j.1365-3083.2005.01686.x 16305646

[46] Bacellar O Brodskyn C Guerreiro J Barral-Netto M Costa CH Coffman RL Interleukin-12 restores interferon-gamma production and cytotoxic responses in visceral leishmaniasis. J Infect Dis (1996) 173(6):1515–8. 10.1093/infdis/173.6.1515 8648233

[47] Maroof A Beattie L Zubairi S Svensson M Stager S Kaye PM. Posttranscriptional regulation of II10 gene expression allows natural killer cells to express immunoregulatory function. Immunity (2008) 29(2):295–305. 10.1016/j.immuni.2008.06.012 18701085 PMC2656759

[48] Prajeeth CK Haeberlein S Sebald H Schleicher U Bogdan C. *Leishmania*-infected macrophages are targets of NK cell-derived cytokines but not of NK cell cytotoxicity. Infect Immun (2011) 79(7):2699–708. 10.1128/IAI.00079-11 21518784 PMC3191990

[49] Haeberlein S Sebald H Bogdan C Schleicher U. IL-18, but not IL-15, contributes to the IL-12-dependent induction of NK-cell effector functions by *Leishmania infantum* in vivo. Eur J Immunol (2010) 40(6):1708–17. 10.1002/eji.200939988 20213736 PMC2909391

[50] Bihl F Pecheur J Breart B Poupon G Cazareth J Julia V Primed antigen-specific CD4+ T cells are required for NK cell activation in vivo upon *Leishmania major* infection. J Immunol (2010) 185(4):2174–81. 10.4049/jimmunol.1001486 20624944

[51] Diefenbach A Schindler H Donhauser N Lorenz E Laskay T MacMicking J Type 1 interferon (IFNalpha/beta) and type 2 nitric oxide synthase regulate the innate immune response to a protozoan parasite. Immunity (1998) 8(1):77–87. 10.1016/S1074-7613(00)80460-4 9462513

[52] Cenini P Berhe N Hailu A McGinnes K Frommel D. Mononuclear cell subpopulations and cytokine levels in human visceral leishmaniasis before and after chemotherapy. J Infect Dis (1993) 168(4):986–93. 10.1093/infdis/168.4.986 8376845

[53] Salaiza-Suazo N Volkow P Tamayo R Moll H Gillitzer R Perez-Torres A Treatment of two patients with diffuse cutaneous leishmaniasis caused by *Leishmania mexicana* modifies the immunohistological profile but not the disease outcome. Trop Med Int Health (1999) 4(12):801–11. 10.1046/j.1365-3156.1999.00491.x 10632987

[54] Pereira LI Dorta ML Pereira AJ Bastos RP Oliveira MA Pinto SA Increase of NK cells and proinflammatory monocytes are associated with the clinical improvement of diffuse cutaneous leishmaniasis after immunochemotherapy with BCG/*Leishmania* antigens. Am J Trop Med Hyg (2009) 81(3):378–83. 19706899

[55] Caneda-Guzman IC Salaiza-Suazo N Fernandez-Figueroa EA Carrada-Figueroa G Aguirre-Garcia M Becker I. NK cell activity differs between patients with localized and diffuse cutaneous leishmaniasis infected with *Leishmania mexicana*: a comparative study of TLRs and cytokines. PLoS One (2014) 9(11):e112410. 10.1371/journal.pone.0112410 25397678 PMC4232367

[56] Fernandez-Figueroa EA Imaz-Rosshandler I Castillo-Fernandez JE Miranda-Ortiz H Fernandez-Lopez JC Becker I Down-regulation of TLR and JAK/STAT pathway genes is associated with diffuse cutaneous leishmaniasis: a gene expression analysis in NK cells from patients infected with *Leishmania mexicana*. PLoS Negl Trop Dis (2016) 10(3):e0004570 10.1371/journal.pntd.0004570 27031998 PMC4816531

[57] Akuffo H Alexis A Eidsmo L Saed A Nylen S Maasho K. Natural killer cells in cross-regulation of IL-12 by IL-10 in *Leishmania* antigen-stimulated blood donor cells. Clin Exp Immunol (1999) 117(3):529–34. 10.1046/j.1365-2249.1999.00994.x 10469058 PMC1905372

[58] Nylen S Maasho K McMahon-Pratt D Akuffo H. Leishmanial amastigote antigen P-2 induces major histocompatibility complex class II-dependent natural killer-cell reactivity in cells from healthy donors. Scand J Immunol (2004) 59(3):294–304. 10.1111/j.0300-9475.2004.01388.x 15030581

[59] Maasho K Satti I Nylen S Guzman G Koning F Akuffo H A *Leishmania* homologue of receptors for activated C-kinase (LACK) induces both interferon-gamma and interleukin-10 in natural killer cells of healthy blood donors. J Infect Dis (2000) 182(2):570–8. 10.1086/315725 10915091

[60] Nylen S Maasho K Soderstrom K Ilg T Akuffo H. Live *Leishmania* promastigotes can directly activate primary human natural killer cells to produce interferon-gamma. Clin Exp Immunol (2003) 131(3):457–67. 10.1046/j.1365-2249.2003.02096.x 12605699 PMC1808651

[61] Becker I Salaiza N Aguirre M Delgado J Carrillo-Carrasco N Kobeh LG *Leishmania* lipophosphoglycan (LPG) activates NK cells through toll-like receptor-2. Mol Biochem Parasitol (2003) 130(2):65–74. 10.1016/S0166-6851(03)00160-9 12946842

[62] Sassi A Largueche-Darwaz B Collette A Six A Laouini D Cazenave PA Mechanisms of the natural reactivity of lymphocytes from noninfected individuals to membrane-associated *Leishmania infantum* antigens. J Immunol (2005) 174(6):3598–607. 10.4049/jimmunol.174.6.3598 15749897

[63] Kurtzhals JA Kemp M Poulsen LK Hansen MB Kharazmi A Theander TG. Interleukin-4 and interferon-gamma production by *Leishmania* stimulated peripheral blood mononuclear cells from nonexposed individuals. Scand J Immunol (1995) 41(4):343–9. 10.1111/j.1365-3083.1995.tb03577.x 7899822

[64] Bogdan C Schonian G Banuls AL Hide M Pratlong F Lorenz E Visceral leishmaniasis in a German child who had never entered a known endemic area: case report and review of the literature. Clin Infect Dis (2001) 32(2):302–6. 10.1086/318476 11170923

[65] Solbach W Forberg K Kammerer E Bogdan C Rollinghoff M. Suppressive effect of cyclosporin A on the development of *Leishmania tropica*-induced lesions in genetically susceptible BALB/c mice. J Immunol (1986) 137(2):702–7. 3487578

[66] Goyard S Segawa H Gordon J Showalter M Duncan R Turco SJ An in vitro system for developmental and genetic studies of *Leishmania donovani* phosphoglycans. Mol Biochem Parasitol (2003) 130(1):31–42. 10.1016/S0166-6851(03)00142-7 14550894

[67] Leitherer S Clos J Liebler-Tenorio EM Schleicher U Bogdan C Soulat D. Characterization of the protein tyrosine phosphatase LmPRL-1 secreted by *Leishmania major* via the exosome pathway. Infect Immun (2017) 85(8). 10.1128/IAI.00084-17 28507071 PMC5520432

[68] Heidkamp GF Sander J Lehmann CHK Heger L Eissing N Baranska A Human lymphoid organ dendritic cell identity is predominantly dictated by ontogeny, not tissue microenvironment. Sci Immunol (2016) 1(6). 10.1126/sciimmunol.aai7677 28783692

[69] Ziegler-Heitbrock L Ancuta P Crowe S Dalod M Grau V Hart DN Nomenclature of monocytes and dendritic cells in blood. Blood (2010) 116(16):e74–80. 10.1182/blood-2010-02-258558 20628149

[70] Stonier SW Schluns KS. Trans-presentation: a novel mechanism regulating IL-15 delivery and responses. Immunol Lett (2010) 127(2):85–92. 10.1016/j.imlet.2009.09.009 19818367 PMC2808451

[71] Bellora F Castriconi R Doni A Cantoni C Moretta L Mantovani A M-CSF induces the expression of a membrane-bound form of IL-18 in a subset of human monocytes differentiating in vitro toward macrophages. Eur J Immunol (2012) 42(6):1618–26. 10.1002/eji.201142173 22678914

[72] Mattiola I Pesant M Tentorio PF Molgora M Marcenaro E Lugli E Priming of human resting NK cells by autologous M1 macrophages via the engagement of IL-1beta, IFN-beta, and IL-15 pathways. J Immunol (2015) 195(6):2818–28. 10.4049/jimmunol.1500325 26276870

[73] Rabinowich H Sedlmayr P Herberman RB Whiteside TL. Response of human NK cells to IL-6 alterations of the cell surface phenotype, adhesion to fibronectin and laminin, and tumor necrosis factor-alpha/beta secretion. J Immunol (1993) 150(11):4844–55. 8496590

[74] Laprevotte E Ysebaert L Klein C Valleron W Blanc A Gross E Endogenous IL-8 acts as a CD16 co-activator for natural killer-mediated anti-CD20 B cell depletion in chronic lymphocytic leukemia. Leuk Res (2013) 37(4):440–6. 10.1016/j.leukres.2012.11.015 23259986

[75] Cai G Kastelein RA Hunter CA. IL-10 enhances NK cell proliferation, cytotoxicity and production of IFN-gamma when combined with IL-18. Eur J Immunol (1999) 29(9):2658–65. 10.1002/(SICI)1521-4141(199909)29:09<2658::AID-IMMU2658>3.0.CO;2-G 10508240

[76] Mocellin S Panelli M Wang E Rossi CR Pilati P Nitti D IL-10 stimulatory effects on human NK cells explored by gene profile analysis. Genes Immun (2004) 5(8):621–30. 10.1038/sj.gene.6364135 15573087

[77] al-Aoukaty A Giaid A Maghazachi AA IL-8 induces calcium mobilization in interleukin-2-activated natural killer cells independently of inositol 1,4,5 trisphosphate. Ann N Y Acad Sci (1995) 766:292–5. 10.1111/j.1749-6632.1995.tb26680.x 7486675

[78] Loetscher P Seitz M Clark-Lewis I Baggiolini M Moser B. Activation of NK cells by CC chemokines. Chemotaxis, Ca2+ mobilization, and enzyme release. J Immunol (1996) 156(1):322–7. 8598480

[79] McDowell MA Marovich M Lira R Braun M Sacks D. *Leishmania* priming of human dendritic cells for CD40 ligand-induced interleukin-12p70 secretion is strain and species dependent. Infect Immun (2002) 70(8):3994–4001. 10.1128/IAI.70.8.3994-4001.2002 12117904 PMC128119

[80] Liese J Schleicher U Bogdan C. TLR9 signaling is essential for the innate NK cell response in murine cutaneous leishmaniasis. Eur J Immunol (2007) 37(12):3424–34. 10.1002/eji.200737182 18034422

[81] Bock E Edvardsen K Gibson A Linnemann D Lyles JM Nybroe O. Characterization of soluble forms of NCAM. FEBS Lett (1987) 225(1–2):33–6. 10.1016/0014-5793(87)81126-2 2446924

[82] Olsen M Krog L Edvardsen K Skovgaard LT Bock E. Intact transmembrane isoforms of the neural cell adhesion molecule are released from the plasma membrane. Biochem J (1993) 295(Pt 3):833–40. 10.1042/bj2950833 8240299 PMC1134637

[83] Semino C Angelini G Poggi A Rubartelli A. NK/iDC interaction results in IL-18 secretion by DCs at the synaptic cleft followed by NK cell activation and release of the DC maturation factor HMGB1. Blood (2005) 106(2):609–16. 10.1182/blood-2004-10-3906 15802534

[84] Hassani K Olivier M. Immunomodulatory impact of *Leishmania*-induced macrophage exosomes: a comparative proteomic and functional analysis. PLoS Negl Trop Dis (2013) 7(5):e2185. 10.1371/journal.pntd.0002185 23658846 PMC3642089

[85] Lieke T Nylen S Eidsmo L McMaster WR Mohammadi AM Khamesipour A *Leishmania* surface protein gp63 binds directly to human natural killer cells and inhibits proliferation. Clin Exp Immunol (2008) 153(2):221–30. 10.1111/j.1365-2249.2008.03687.x 18713141 PMC2492898

[86] Meddeb-Garnaoui A Zrelli H Dellagi K. Effects of tropism and virulence of *Leishmania* parasites on cytokine production by infected human monocytes. Clin Exp Immunol (2009) 155(2):199–206. 10.1111/j.1365-2249.2008.03821.x 19040614 PMC2675250

[87] Gregory DJ Sladek R Olivier M Matlashewski G. Comparison of the effects of *Leishmania major* or *Leishmania donovani* infection on macrophage gene expression. Infect Immun (2008) 76(3):1186–92. 10.1128/IAI.01320-07 18086813 PMC2258831

[88] Ziegler-Heitbrock L. Monocyte subsets in man and other species. Cell Immunol (2014) 289(1–2):135–9. 10.1016/j.cellimm.2014.03.019 24791698

[89] Ferlazzo G Pack M Thomas D Paludan C Schmid D Strowig T Distinct roles of IL-12 and IL-15 in human natural killer cell activation by dendritic cells from secondary lymphoid organs. Proc Natl Acad Sci U S A (2004) 101(47):16606–11. 10.1073/pnas.0407522101 15536127 PMC534504

[90] Carson WE Dierksheide JE Jabbour S Anghelina M Bouchard P Ku G Coadministration of interleukin-18 and interleukin-12 induces a fatal inflammatory response in mice: critical role of natural killer cell interferon-gamma production and STAT-mediated signal transduction. Blood (2000) 96(4):1465–73. 10942393

[91] Smyth MJ Swann J Kelly JM Cretney E Yokoyama WM Diefenbach A NKG2D recognition and perforin effector function mediate effective cytokine immunotherapy of cancer. J Exp Med (2004) 200(10):1325–35. 10.1084/jem.20041522 15545356 PMC2211920

[92] Lauwerys BR Renauld JC Houssiau FA. Synergistic proliferation and activation of natural killer cells by interleukin 12 and interleukin 18. Cytokine (1999) 11(11):822–30. 10.1006/cyto.1999.0501 10547269

[93] Takeda K Tsutsui H Yoshimoto T Adachi O Yoshida N Kishimoto T Defective NK cell activity and Th1 response in IL-18-deficient mice. Immunity (1998) 8(3):383–90. 10.1016/S1074-7613(00)80543-9 9529155

[94] Zahn S Kirschsiefen P Jonuleit H Steinbrink K Von Stebut E. Human primary dendritic cell subsets differ in their IL-12 release in response to *Leishmania major* infection. Exp Dermatol (2010) 19(10):924–6. 10.1111/j.1600-0625.2010.01149.x 20707811

[95] Haniffa M Gunawan M Jardine L. Human skin dendritic cells in health and disease. J Dermatol Sci (2015) 77(2):85–92. 10.1016/j.jdermsci.2014.08.012 25301671 PMC4728191

[96] Lugini L Cecchetti S Huber V Luciani F Macchia G Spadaro F Immune surveillance properties of human NK cell-derived exosomes. J Immunol (2012) 189(6):2833–42. 10.4049/jimmunol.1101988 22904309

[97] Ziegler S Weiss E Schmitt AL Schlegel J Burgert A Terpitz U CD56 is a pathogen recognition receptor on human natural killer cells. Sci Rep (2017) 7(1):6138. 10.1038/s41598-017-06238-4 28733594 PMC5522490

[98] Mace EM Gunesch JT Dixon A Orange JS. Human NK cell development requires CD56-mediated motility and formation of the developmental synapse. Nat Commun (2016) 7:12171. 10.1038/ncomms12171 27435370 PMC4961740

[99] Valgardsdottir R Capitanio C Texido G Pende D Cantoni C Pesenti E Direct involvement of CD56 in cytokine-induced killer-mediated lysis of CD56+ hematopoietic target cells. Exp Hematol (2014) 42(12):1013–21.e1. 10.1016/j.exphem.2014.08.005 25201755

