# Supplementary material for: Monocyte-Derived Signals Activate Human Natural Killer Cells in Response to Leishmania Parasites
Source: Front Immunol. 2018 Jan 24;9:24. doi: 10.3389/fimmu.2018.00024 (PMC5810259; doi:10.3389/fimmu.2018.00024)
Supplement: Supplementary file 1 [file Table_1.DOCX]

**Supplementary Table 1**: IL-18 content (pg/ml) in cell culture supernatants of purified monocyte/*Leishmania* 20 h cocultures measured by ELISA

| **Experiment** | **Medium** | ***L. major*** | ***L. infantum*** | ***L. mexicana*** |
| --- | --- | --- | --- | --- |
| **1** | nd | nd | nd | nd |
| **2** | nd | nd | 37.8 | nd |
| **3** | nd | 413.6 | nd | nd |
| **4** | nd | nd | nd | nd |
| **5** | nd | 92.2 | nd | nd |
| **6** | nd | 456.8 | nd | nd |
| **7** | nd | nd | nd | not done |
| **8** | nd | nd | nd | not done |
| **9** | nd | nd | not done | not done |
| **10** | nd | nd | not done | not done |

nd = not detectable
