# Supplementary material for: Monocyte-Derived Signals Activate Human Natural Killer Cells in Response to Leishmania Parasites
Source: Front Immunol. 2018 Jan 24;9:24. doi: 10.3389/fimmu.2018.00024 (PMC5810259; doi:10.3389/fimmu.2018.00024)
Supplement: Supplementary file 2 [file Table_2.DOCX]

**Supplementary Table 2:** Cytokines/chemokines (pg/ml) produced during 20 h coculture of purified NK cells and monocytes together with *L. major*. Measurement was done with a ProcartaPlex^®^ Multiplex Immunoassay.

| **Cytokines/chemokines**  **pg/ml** | **Donor 1** | **Donor 2** | **Donor 3** | **Mean ± SD** |
| --- | --- | --- | --- | --- |
| **BDNF** | <1.721 | <1.721 | <1.721 |  |
| **EGF** | <2.588 | <2.588 | <2.588 |  |
| **Eotaxin** | <0.842 | <0.842 | <0.842 |  |
| **FGF-2** | <3.027 | <3.027 | <3.027 |  |
| **GM-CSF** | <20.874 | <20.874 | <20.874 |  |
| **GRO alpha** | 146.762 | 49.457 | <1.55 | 91.1 ± 68.8 |
| **HGF** | <8.789 | <8.789 | <8.789 |  |
| **IFN alpha** | <0.632 | <0.632 | <0.632 |  |
| **IFN gamma** | <6.006 | <6.006 | <6.006 |  |
| **IL-1RA** | 17,880.908 | 10,091.281 | 913.279 | 9,628.5±8493.3 |
| **IL-1 alpha** | 1.888 | <0.623 | <0.623 |  |
| **IL-1 beta** | 322.467 | 323.573 | 21.189 | 222.4±174.3 |
| **IL-2** | <4.956 | <4.956 | <4.956 |  |
| **IL-4** | 35.311 | <14.551 | <14.551 |  |
| **IL-5** | <6.787 | <6.787 | <6.787 |  |
| **IL-6** | 717.619 | 101.299 | 11.044 | 276.7±384.5 |
| **IL-7** | <0.586 | <0.586 | <0.586 |  |
| **IL-8** | 3,316.978 | 13,704.459 | 1,411.216 | 6,144.2±6616.3 |
| **IL-9** | <10.01 | <10.01 | 23.583 |  |
| **IL-10** | 1.628 | 1.556 | <1.489 | 1.6±0.1 |
| **IL-12p70** | <6.738 | <6.738 | <6.738 |  |
| **IL-13** | <2.002 | <2.002 | <2.002 |  |
| **IL-15** | <2.954 | <2.954 | <2.954 |  |
| **IL-17A** | <2.441 | <2.441 | <2.441 |  |
| **IL-18** | 61.005 | 51.233 | <9.77 | 56.1±6.9 |
| **IL-21** | 38.167 | <10.596 | <10.596 |  |
| **IL-22** | <29.98 | <29.98 | <29.98 |  |
| **IL-23** | <10.254 | <10.254 | <10.254 |  |
| **IL-27** | <21.729 | <21.729 | <21.729 |  |
| **IL-31** | <22.583 | <22.583 | <22.583 |  |
| **IP-10** | <2.563 | <2.563 | <2.563 |  |
| **LIF** | 16.968 | <5.347 | <5.347 |  |
| **MCP-1** | 83.526 | 19.022 | <1.746 | 51.3± 45.6 |
| **MIP-1 alpha** | >5,950 | 196.189 | 49.553 | 2,065.2±3,365.1 |
| **MIP-1 beta** | 3,735.44 | 1,924.159 | 614.766 | 2,091.5±1567 |
| **NGF beta** | <7.935 | <7.935 | <7.935 |  |
| **RANTES** | <0.598 | 29.232 | <0.598 |  |
| **PDGF-BB** | 2,678.69 | 288.481 | <7.568 | 1,483.6±1,690.1 |
| **PIGF-1** | 3.321 | 1.356 | <1.123 |  |
| **SCF** | <1.477 | <1.477 | 1.842 |  |
| **SDF-1 alpha** | 701.21 | 384.023 | <13.55 | 542.6±224.3 |
| **TNF alpha** | 40.358 | <4.736 | <4.736 |  |
| **TNF beta** | <3.54 | <3.54 | <3.54 |  |
| **VEGF-A** | 56.153 | 173.374 | 272.126 | 167.2±108.1 |
| **VEGF-D** | <8.74 | <8.74 | <8.74 |  |

Values with a less than symbol (<) denote the cytokine-specific detection threshold.
