# Supplementary material for: Monocyte-Derived Signals Activate Human Natural Killer Cells in Response to Leishmania Parasites
Source: Front Immunol. 2018 Jan 24;9:24. doi: 10.3389/fimmu.2018.00024 (PMC5810259; doi:10.3389/fimmu.2018.00024)
Supplement: Supplementary file 3 [file Image_1.PDF]

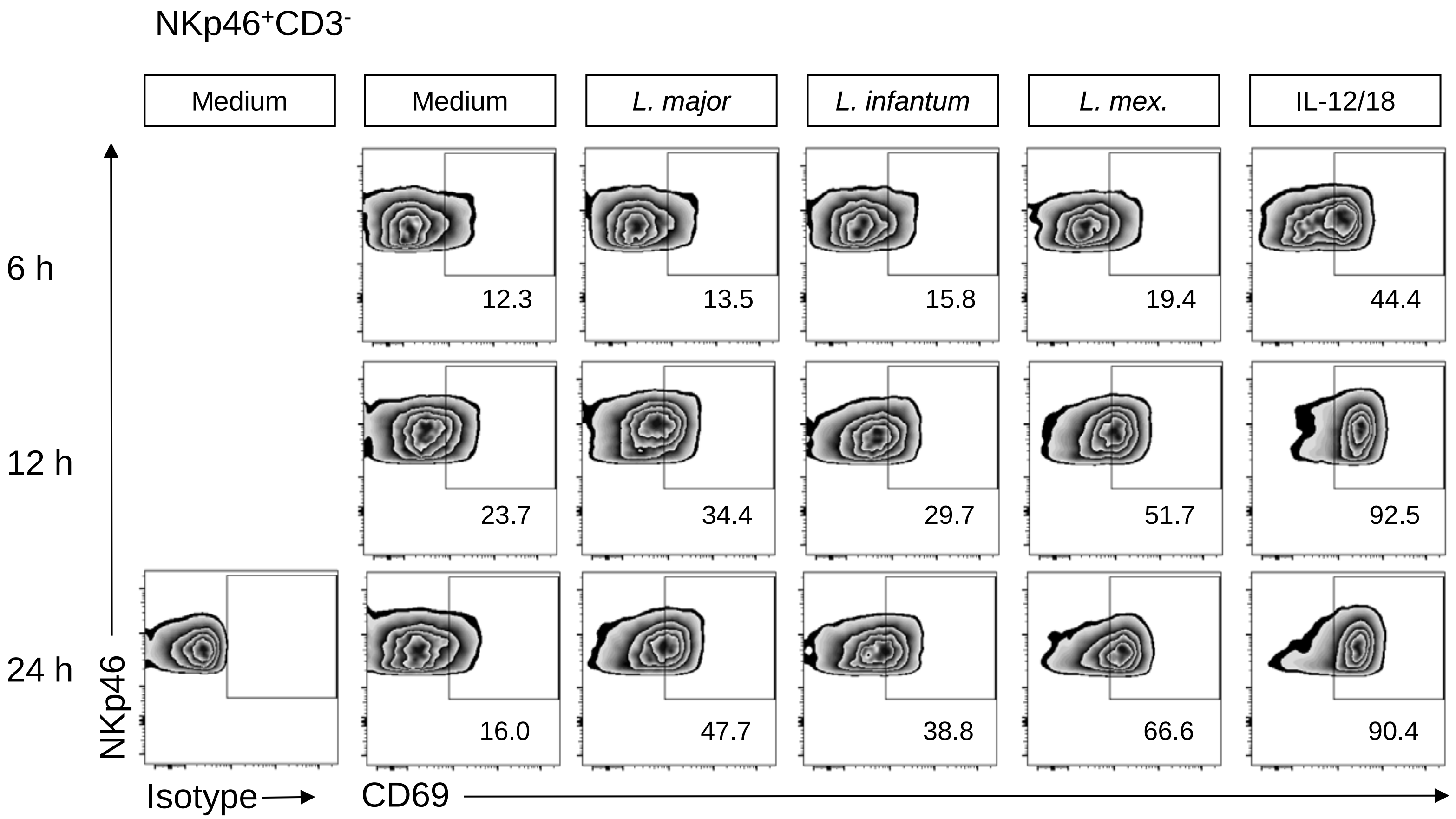

Suppl. Fig. 1: Human PBMCs were co-cultured for 20h with *Leishmania* promastigotes of different species (MOI 10) or IL-12 plus IL-18 (10 ng/ml). After 6, 12 and 20h of incubation surface expression of CD69 on NKp46^{+}CD3^{-} NK cells was analysed by flow cytometry. Flow cytometry plots show representative results of NKp46^{+}CD3^{-} NK cells of one out of three different blood donors tested.
